# Supplementary material for: Digital twin for sex-specific identification of class III antiarrhythmic drugs based on in vitro measurements, computer models, and machine learning tools
Source: PLoS Comput Biol. 2025 Jul 3;21(7):e1013154. doi: 10.1371/journal.pcbi.1013154 (PMC12510667; doi:10.1371/journal.pcbi.1013154)
Supplement: S1 Text — (DOCX) [file pcbi.1013154.s001.docx]

# S1_Text: Feature importance analysis.

**Fig A.** Feature analysis of sex-specific classifiers. Feature importance for the male (A) and female (B) classifiers is measured by the SHAP value. Feature correlation analysis shows that the two key features have a high degree of negative correlation (C).
