# Supplementary material for: Digital twin for sex-specific identification of class III antiarrhythmic drugs based on in vitro measurements, computer models, and machine learning tools
Source: PLoS Comput Biol. 2025 Jul 3;21(7):e1013154. doi: 10.1371/journal.pcbi.1013154 (PMC12510667; doi:10.1371/journal.pcbi.1013154)
Supplement: S2 Text — (DOCX) [file pcbi.1013154.s002.docx]

# S2_Text: Patient characteristics.

**Table A.** Patient characteristics ^1^.

| **Patient characteristics** | **AF patients (*n*=201)** |
| --- | --- |
| Age (years) | 72.5 ± 7.3 |
| Sex, male *n* (%) | 126 (62.7) |
| Body mass index kg/m^2^ | 28.0 ± 4.3 |
| Hypertension *n* (%) | 192 (97.0) |
| Diabetes mellitus *n* (%) | 79 (39.9) |
| Hyperlipidemia *n* (%) | 130 (70.3) |
| Coronary artery disease *n* (%) | 94 (46.8) |
| Valvular disease *n* (%) | 175 (87.1) |
| Left ventricular ejection fraction (%) | 52.1 ± 11.9 |
| Left ventricular end diastolic diameter (mm) | 51.9 ± 7.7 |
| Left ventricular end diastolic pressure (mm Hg) | 51.2 ± 8.2 |
| Left atrial diameter (mm) | 51.2 ± 8.2 |
| Left ventricular hypertrophy *n* (%) | 119 (63.6) |
| Beta-blockers *n* (%) | 173 (86.5) |
| Digitalis *n* (%) | 61 (30.3) |
| Ca^2+^-channel-blockers *n* (%) | 43 (21.7) |
| ACE-inhibitors *n* (%) | 130 (66.0) |
| AT_1_-blockers *n* (%) | 54 (27.3) |
| Diuretics *n* (%) | 152 (76.8) |
| Statins *n* (%) | 110 (55.8) |
| Nitrates *n* (%) | 21 (10.6) |

1. Pecha, S. *et al.* Resting membrane potential is less negative in trabeculae from right atrial appendages of women, but action potential duration does not shorten with age. J Mol Cell Cardiol **176**, 1-10 (2023).
