# Supplementary material for: Digital twin for sex-specific identification of class III antiarrhythmic drugs based on in vitro measurements, computer models, and machine learning tools
Source: PLoS Comput Biol. 2025 Jul 3;21(7):e1013154. doi: 10.1371/journal.pcbi.1013154 (PMC12510667; doi:10.1371/journal.pcbi.1013154)
Supplement: S3 Text — (DOCX) [file pcbi.1013154.s003.docx]

# S3_Text: Values of biomarkers of action potential recordings from AF patients (male vs. female).

**Table A.** Values of biomarkers of action potential recordings from AF patients (male vs. female).

| **AP biomarkers** | **Male (*n*=180)** | **Female (*n*=107)** |
| --- | --- | --- |
| RMP (mV) | -77.57 ± 0.30 | -77.27 ± 0.39 |
| dV/dt_max_ (V/S) | 248.23 ± 5.65 | 233.1 ± 7.28 |
| APA (mV) | 102.20 ± 0.59 | 100.98 ± 0.77 |
| APD_20_ (ms) | 28.03 ± 1.15 | 29.00 ± 1.48 |
| APD_50_ (ms) | 99.19 ± 3.09 | 101.79 ± 3.98 |
| APD_90_(ms) | 216.01 ± 3.34 | 222.19 ± 4.30 |
| V20 (mV) | 5.56 ± 0.72 | 6.63 ± 0.93 |
