# Supplementary material for: Digital twin for sex-specific identification of class III antiarrhythmic drugs based on in vitro measurements, computer models, and machine learning tools
Source: PLoS Comput Biol. 2025 Jul 3;21(7):e1013154. doi: 10.1371/journal.pcbi.1013154 (PMC12510667; doi:10.1371/journal.pcbi.1013154)
Supplement: S4 Text — (DOCX) [file pcbi.1013154.s004.docx]

# S4_Text: The flowchart illustrates the process of constructing AF populations of males and females.


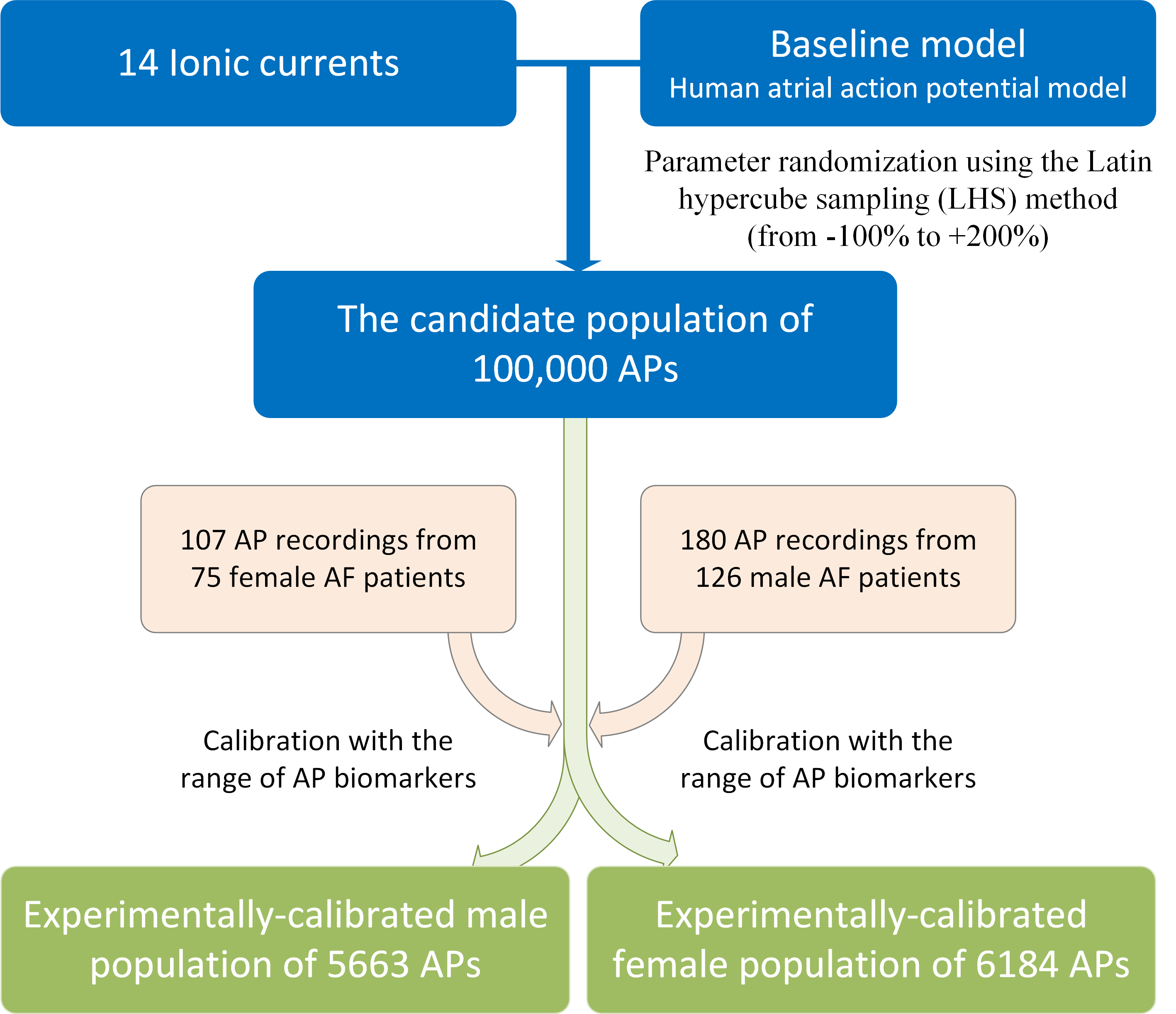


**Fig A.** The flowchart illustrates the process of constructing AF populations of males and females. AP, action potential.
