# Supplementary material for: Digital twin for sex-specific identification of class III antiarrhythmic drugs based on in vitro measurements, computer models, and machine learning tools
Source: PLoS Comput Biol. 2025 Jul 3;21(7):e1013154. doi: 10.1371/journal.pcbi.1013154 (PMC12510667; doi:10.1371/journal.pcbi.1013154)
Supplement: S6 Text — (DOCX) [file pcbi.1013154.s006.docx]

# S6_Text: Histograms of the [scaling factors](https://www.sciencedirect.com/topics/computer-science/scaling-factor) for the different ionic currents of the selected individuals (5,663 males and 6,184 females).


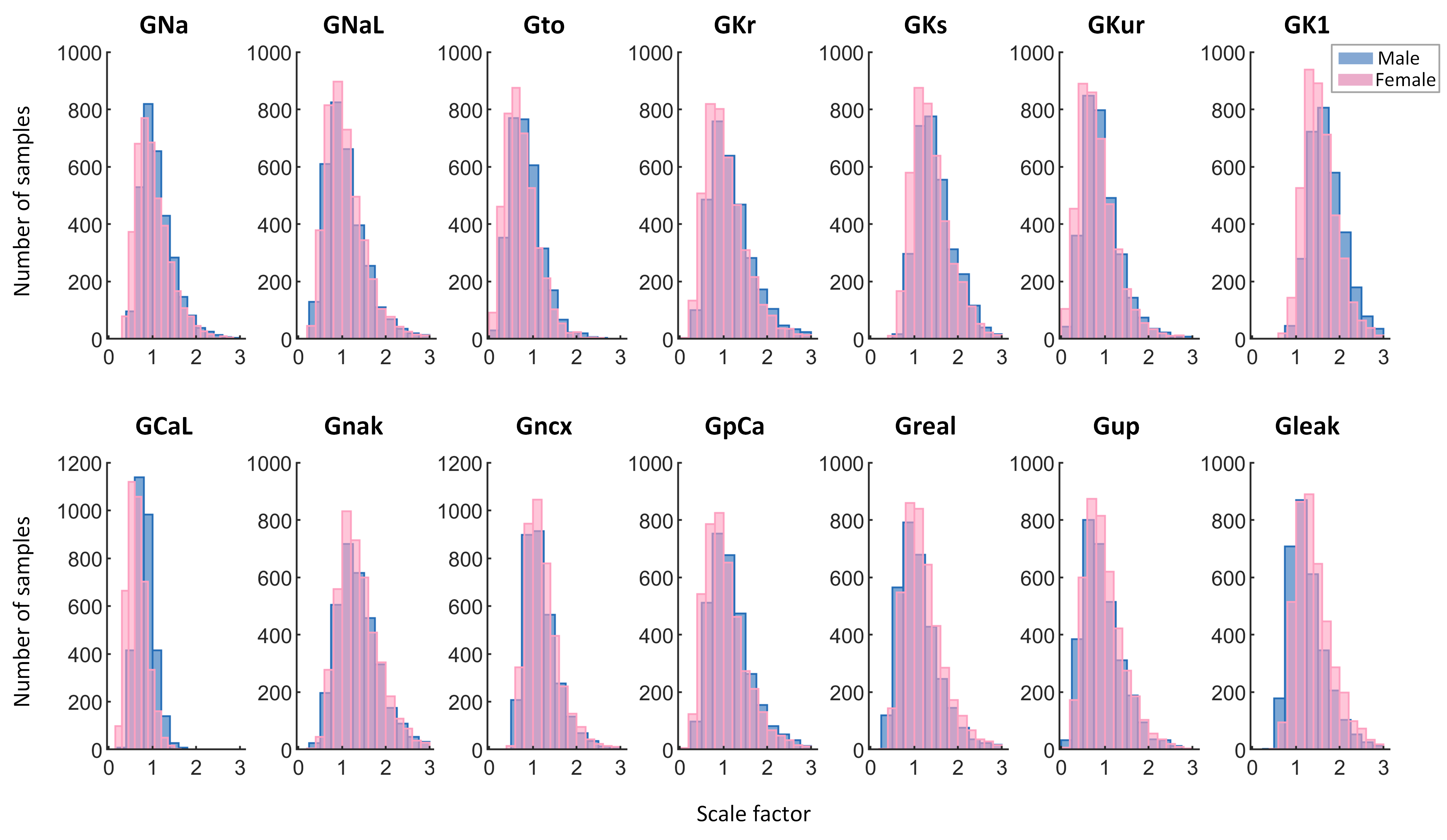


**Fig A.** Histograms of the [scaling factors](https://www.sciencedirect.com/topics/computer-science/scaling-factor) for the different ionic currents of the selected individuals (5,663 males and 6,184 females). Male population is represented in blue and female population is represented in pink.
