# Supplementary material for: Digital twin for sex-specific identification of class III antiarrhythmic drugs based on in vitro measurements, computer models, and machine learning tools
Source: PLoS Comput Biol. 2025 Jul 3;21(7):e1013154. doi: 10.1371/journal.pcbi.1013154 (PMC12510667; doi:10.1371/journal.pcbi.1013154)
Supplement: S7 Text — (DOCX) [file pcbi.1013154.s007.docx]

# S7_Text: The list of antiarrhythmic drugs with their concentration(μM), IC50 (μM) and hill coefficient.

**Table A.** The list of antiarrhythmic drugs with their concentration(μM), IC50 (μM) and hill coefficient ^1,2^.

| **Drugs** | | **Concentration**  **(μM)** | **IC 50 values (hill coefficient)** | | | | | | | | | |
| --- | --- | --- | --- | --- | --- | --- | --- | --- | --- | --- | --- | --- |
|  |  |  | INa | INaL | Ito | ICaL | IKur | IKr | IKs | IK1 | INaCa | INaK |
| **Class Ⅲ** | Amiodarone | 2.0 | 5  (1.0) | - | 3.8  (0.4) | 1.5  (0.6) | - | 3.0  (1.0) | 100  (1.0) | - | 3.4  (1.0) | - |
|  | Dofetilide | 0.002 | 0.3805  (0.9) | 753.16 (0.3) | 0.0188  (0.8) | 0.263  (1.3) | - | 0.0049  (0.9) | - | 0.3943  (0.8) | - | - |
|  | Dronedarone | 0.205 | 0.54  (2.03) | - | - | 0.83  (2.75) | 1.0 (1.0) | 0.0591  (0.8) | 5.6  (0.51) | - | - | - |
|  | Ibutilide | 0.015 | - | - | - | - | - | 0.02  (1.0) | - | - | - | - |
|  | Sotalol | 14.69 | 1140000 (0.5) | - | 43143.55 (0.7) | 7061.527 (0.9) | - | 110.6  (0.8) | 4221.856 (1.2) | 3050.26  (1.2) | - | - |
|  | Vernakalant | 30 | 90  (1.0) | - | 15  (1.0) | 84  (1.0) | 15  (1.0) | 20  (1.0) | - | - | - | - |
| **Non-Class Ⅲ** | Digoxin | 0.04 | - | - | - | - | - | 0.054  (1.0) | - | - | - | 0.12 (1.0) |
|  | Disopyramide | 0.742 | 168.4  (1.0) | - | - | 1036.7  (1.0) | - | 14.4  (1) | - | - | - | - |
|  | Flecainide | 2.5 | 6.5  (1.0) | - | 10.0  (0.8) | 27.1  (1.0) | 2.9 (1.0) | 1.6  (1.0) | - | - | - | - |
|  | Propafenone | 0.8 | 2.5  (1.0) | - | 7.2  (1.0) | 1.55  (1.0) | - | 0.44  (1.0) | 16  (1.0) | - | - | - |
|  | Quinidine | 3.237 | 12.329  (1.5) | 9.417  (1.3) | 3.4874  (1.3) | 51.5923 (0.6) | - | 0.992  (0.8) | 4.8989  (1.4) | 39589.919 (0.4) |  |  |
|  | Ranolazine | 10 | 200  (1.0) | - | - | 250  (1.0) | - | 12  (1.0) | 100  (1.0) | - | 91 (1.0) | - |

1. Fogli Iseppe, A. *et al.* Sex-Specific Classification of Drug-Induced Torsade de Pointes Susceptibility Using Cardiac Simulations and Machine Learning. Clin Pharmacol Ther **110**, 380-391 (2021).
2. Dasí, A. *et al.* In-silico drug trials for precision medicine in atrial fibrillation: From ionic mechanisms to electrocardiogram-based predictions in structurally-healthy human atria. Front. physiol. **13**, 966046 (2022).
