# Supplementary material for: Digital twin for sex-specific identification of class III antiarrhythmic drugs based on in vitro measurements, computer models, and machine learning tools
Source: PLoS Comput Biol. 2025 Jul 3;21(7):e1013154. doi: 10.1371/journal.pcbi.1013154 (PMC12510667; doi:10.1371/journal.pcbi.1013154)
Supplement: S8 Text — (DOCX) [file pcbi.1013154.s008.docx]

# 8_Text: the distribution of drug populations.


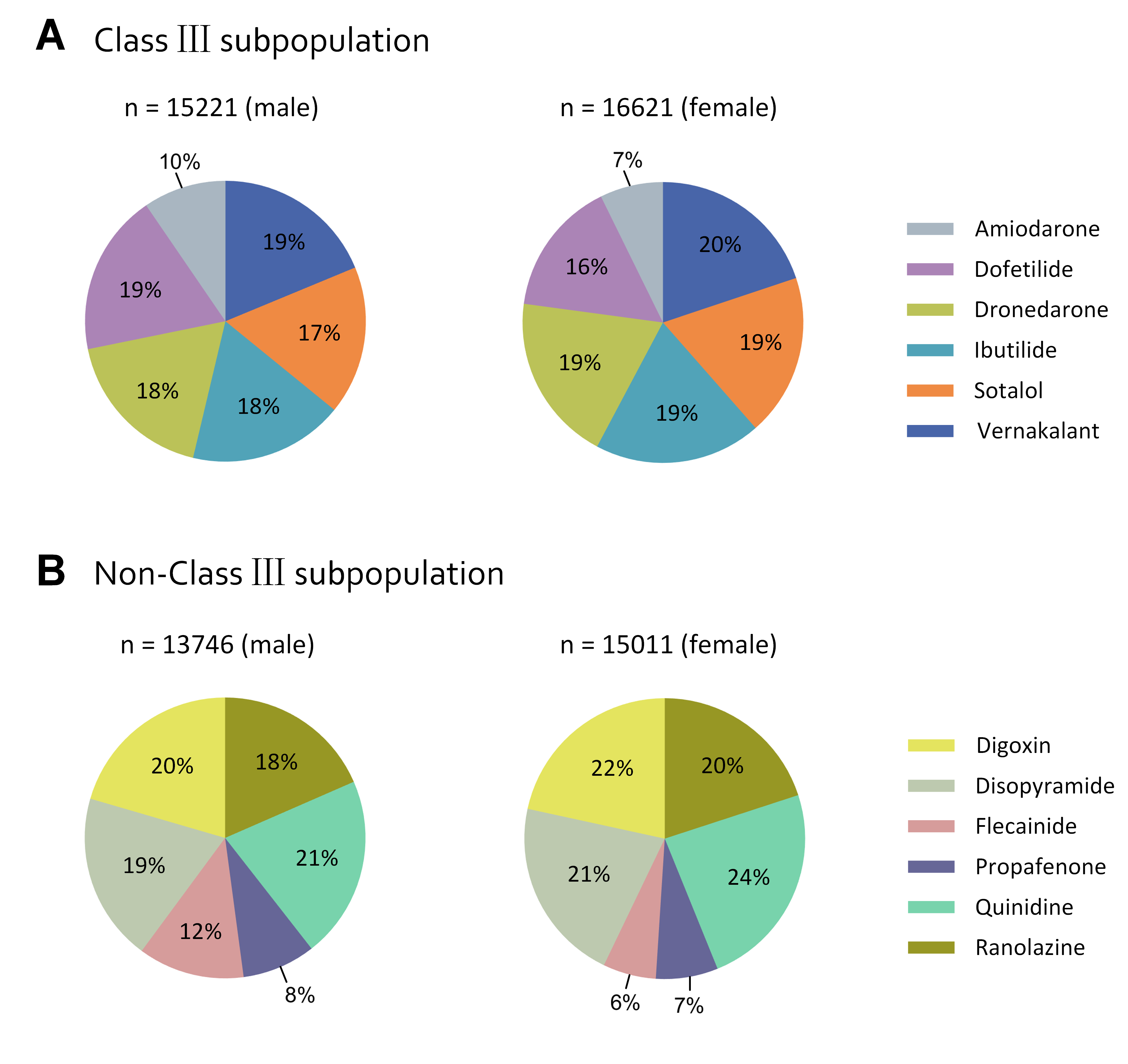


**Fig A.** illustrated the distribution of drug populations. **(A)** detailed the Class III drug population of males, comprising 15,221 action potentials (APs). The distribution of these drugs was as follows: Amiodarone (10%), Dofetilide (19%), Dronedarone (18%), Ibutilide (18%), Sotalol (17%), and Vernakalant (19%). Conversely, the Class III drug population of females included 16,621 APs, with the following proportions: Amiodarone (7%), Dofetilide (16%), Dronedarone (19%), Ibutilide (19%), Sotalol (20%), and Vernakalant (19%). **(B)** presents the non-Class III drug population of males, totaling 13,746 APs. The composition of this group was: Digoxin (20%), Disopyramide (19%), Flecainide (12%), Propafenone (8%), Quinidine (21%), and Ranolazine (18%). Moreover, the non-Class III drug population for females, consisting of 15,011 APs, is distributed as follows: Digoxin (22%), Disopyramide (21%), Flecainide (6%), Propafenone (7%), Quinidine (24%), and Ranolazine (20%).
