# Supplementary material for: Digital twin for sex-specific identification of class III antiarrhythmic drugs based on in vitro measurements, computer models, and machine learning tools
Source: PLoS Comput Biol. 2025 Jul 3;21(7):e1013154. doi: 10.1371/journal.pcbi.1013154 (PMC12510667; doi:10.1371/journal.pcbi.1013154)
Supplement: S9 Text — (DOCX) [file pcbi.1013154.s009.docx]

# S9_Text: The model screening process of constructing drug populations of males and females.

**Table A.** The model screening process of constructing drug populations of males and females.

| Drug Class | Drug name | Initial population | Abnormal repolarization | Final population | Drug population |
| --- | --- | --- | --- | --- | --- |
| non-Class Ⅲ | Amiodarone | 11,847  (male: 5,663 vs. female: 6,184) | 9,162  (male: 4,141 vs female: 5,021) | 2,685  (male: 1,522 vs female: 1,163) | 31,842  (male:15,221 vs female:16,621) |
|  | Dofetilide | 11,847  (male: 5,663 vs. female: 6,184) | 5,551  (male: 2,892 vs female: 2,659) | 6,296  (male: 2,771 vs female: 3,525) |  |
|  | Dronedarone | 11,847  (male: 5,663 vs. female: 6,184) | 6,098  (male:3,075 vs female:3,023) | 5,749  (male:2,588 vs female:3,161) |  |
|  | Ibutilide | 11,847  (male: 5,663 vs. female: 6,184) | 5,950  (male:2,923 vs female:3,027) | 5,897  (male:2,740 vs female:3,157) |  |
|  | Sotalol | 11,847  (male: 5,663 vs. female: 6,184) | 6,103  (male:3,076 vs female:3,027) | 5,744  (male:2,587 vs female:3,157) |  |
|  | Vernakalant | 11,847  (male: 5,663 vs. female: 6,184) | 5,631  (male:2,771 vs female:2,860) | 6,216  (male:2,892 vs female:3,324) |  |
| non-Class Ⅲ | Digoxin | 11,847  (male: 5,663 vs. female: 6,184) | 5,796  (male:2,914 vs female:2,882) | 6,051  (male:2,749 vs female:3,302) | 28,757  (male: 13,746 vs female: 15,011) |
|  | Disopyramide | 11,847  (male: 5,663 vs. female: 6,184) | 6,084  (male:3,052 vs female:3,032) | 5,763  (male:2,611 vs female:3,152) |  |
|  | Flecainide | 11,847  (male: 5,663 vs. female: 6,184) | 9,159  (male:3,876 vs female:5,283) | 2,688  (male:1,787 vs female:901) |  |
|  | Propafenone | 11,847  (male: 5,663 vs. female: 6,184) | 9,559  (male:4,426 vs female:5,133) | 2,288  (male:1,237 vs female:1,051) |  |
|  | Quinidine | 11,847  (male: 5,663 vs. female: 6,184) | 5,359  (male:2,777 vs female:2,582) | 6,488  (male:2,886 vs female:3,602) |  |
|  | Ranolazine | 11,847  (male: 5,663 vs. female: 6,184) | 6,368  (male:3,187 vs female:3,181) | 5,479  (male:2,476 vs female:3,003) |  |
