# Supplementary material for: Digital twin for sex-specific identification of class III antiarrhythmic drugs based on in vitro measurements, computer models, and machine learning tools
Source: PLoS Comput Biol. 2025 Jul 3;21(7):e1013154. doi: 10.1371/journal.pcbi.1013154 (PMC12510667; doi:10.1371/journal.pcbi.1013154)
Supplement: S10 Text — (DOCX) [file pcbi.1013154.s010.docx]

# S10_Text: Values of biomarkers of the AAD population (Class III vs. non-Class III).

**Table A.** Values of biomarkers of the AAD population (Class III vs. non-Class III). Statistical significance: *p<0.05; **p<0.01; and ***p<0.001.

|  | | **RMP (mV)** | **dV/dt_max_**  **(V/s)** | **APA**  **(mV)** | **APD_20_（ms）** | **APD_40_**  **（ms）** | **APD_50_**  **（ms）** | **APD_90_**  **（ms）** | **APD_tri_**  **（ms）** | **CTA**  **(nM)** | **CT_max_**  **(nM)** | **CTD_50_**  **(ms)** | **CTD_90_**  **(ms)** | **CTD_tri_**  **(ms)** | **CTD**  **(nM)** |
| --- | --- | --- | --- | --- | --- | --- | --- | --- | --- | --- | --- | --- | --- | --- | --- |
| **Class Ⅲ** | Amiodarone | -76.10  ±1.12 | 155.00  ±22.15 | 114.88  ±3.03 | 7.40  ±0.87 | 70.04  ±10.53 | 106.08  ±10.12 | 260.33  ±14.77 | 154.25  ±12.57 | 211.81  ±77.21 | 437.58  ±82.11 | 256.90  ±41.93 | 561.57  ±70.20 | 304.67  ±30.85 | 228.15  ±17.11 |
|  | Dofetilide | -75.86  ±1.26 | 192.31  ±33.53 | 115.22  ±3.35 | 6.02  ±0.77 | 94.09  ±19.27 | 134.71  ±17.31 | 310.63  ±34.3 | 175.92  ±23.30 | 288.02  ±100.95 | 505.52  ±108.98 | 234.24  ±45.11 | 541.76  ±75.72 | 307.52  ±32.52 | 220.59  ±17.95 |
|  | Dronedarone | -76.38  ±1.12 | 199.32  ±29.39 | 114.92  ±3.22 | 5.68  ±0.60 | 91.28  ±17.76 | 133.85  ±15.50 | 316.96  ±32.06 | 183.11  ±22.58 | 288.21  ±98.21 | 503.99  ±106.57 | 232.40  ±44.52 | 541.26  ±74.79 | 308.86  ±32.06 | 218.89  ±18.06 |
|  | Ibutilide | -75.79  ±1.35 | 192.37  ±36.20 | 113.91  ±3.73 | 5.73  ±0.65 | 94.42  ±18.86 | 134.25  ±17.38 | 313.29  ±35.17 | 179.03  ±23.61 | 244.36  ±88.23 | 456.92  ±93.31 | 248.14  ±42.95 | 565.85  ±68.88 | 317.71  ±28.003 | 215.75  ±16.82 |
|  | Sotalol | -75.76  ±1.24 | 194.77  ±33.05 | 114.48  ±3.39 | 5.81  ±0.68 | 93.98  ±19.21 | 134.94  ±17.30 | 310.87  ±33.74 | 175.93  ±23.04 | 295.36  ±101.87 | 515.59  ±108.36 | 232.45  ±43.54 | 540.57  ±73.06 | 308.12  ±31.29 | 223.39  ±16.54 |
|  | Vernakalant | -75.93  ±1.09 | 187.68  ±22.46 | 117.52  ±2.26 | 6.78  ±0.97 | 100.74  ±18.02 | 139.85  ±16.23 | 311.34  ±29.87 | 171.49  ±20.33 | 275.90  ±101.55 | 494.23  ±108.78 | 241.38  ±47.713 | 546.75  ±77.631 | 305.37  ±32.175 | 221.31  ±17.477 |
|  | All(*n*=31,842) | -75.95  ±1.20 | 187.19  ±33.01 | 115.21  ±3.37 | 6.23  ±4.98 | 91.27  ±19.79 | 131.21  ±18.88 | 304.55  ±34.87 | 173.34  ±22.36 | 270.72  ±100.01 | 489.14  ±106.20 | 239.84  ±45.41 | 547.96  ±74.64 | 308.12  ±31.73 | 221.40  ±17.74 |
| **non-Class Ⅲ** | Digoxin | -75.534  ±1.26 | 187.81  ±35.08 | 113.29  ±3.65 | 5.7131  ±0.63 | 87.25  ±19.54 | 131.72  ±15.75 | 306.54  ±26.34 | 174.82  ±18.97 | 278.61  ±95.35 | 493.70  ±103.16 | 232.55  ±44.13 | 539.73  ±74.17 | 307.18  ±31.80 | 217.99  ±17.44 |
|  | Disopyramide | -75.70  ±1.41 | 184.09  ±42.69 | 113.15  ±4.23 | 5.8114  ±0.72 | 87.94  ±19.64 | 128.65  ±16.75 | 296.9  ±28.78 | 168.25  ±20.27 | 277.94  ±98.07 | 494.40  ±105.76 | 233.28  ±44.02 | 541.70  ±75.08 | 308.42  ±32.83 | 219.45  ±17.75 |
|  | Flecainide | -76.176  ±0.68 | 189.11  ±17.18 | 116.45  ±2.68 | 6.542  ±1.12 | 113.27  ±16.03 | 147.60  ±12.93 | 311.12  ±14.12 | 163.52  ±16.08 | 336.43  ±94.22 | 539.30  ±100.56 | 204.71  ±35.47 | 491.29  ±68.29 | 286.58  ±33.72 | 205.40  ±13.40 |
|  | Propafenone | -76.26  ±1.35 | 169.01  ±35.10 | 113.39  ±3.74 | 6.0927  ±0.76 | 79.81  ±17.14 | 119.34  ±14.73 | 284.51  ±25.87 | 165.18  ±18.199 | 212.25  ±77.92 | 425.52  ±84.84 | 250.84  ±43.96 | 564.28  ±71.34 | 313.45  ±29.64 | 215.87  ±17.12 |
|  | Quinidine | -76.19  ±1.17 | 178.02  ±26.03 | 116.93  ±2.59 | 6.98  ±1.02 | 91.00  ±16.78 | 129.21  ±14.46 | 298.41  ±21.25 | 169.20  ±14.91 | 240.81  ±96.62 | 457.73  ±103.53 | 249.06  ±48.47 | 555.63  ±78.84 | 306.57  ±32.71 | 219.62  ±17.64 |
|  | Ranolazine | -75.95  ±1.36 | 184.37  ±40.58 | 113.44  ±3.98 | 5.79  ±0.68 | 82.45  ±18.41 | 124.63  ±15.67 | 298.50  ±28.12 | 173.87  ±19.72 | 281.96  ±97.87 | 500.79  ±105.35 | 232.09  ±43.10 | 540.08  ±74.17 | 307.99  ±32.83 | 221.83  ±17.94 |
|  | All(*n*=28,757) | -75.92  ±1.35* | 179.92  ±38.58*** | 113.64  ±4.01*** | 5.98  ±0.82** | 84.90  ±18.65* | 125.79  ±15.87** | 295.40  ±26.43*** | 169.61  ±18.70** | 259.43  ±97.33** | 475.54  ±104.81 | 238.62  ±45.07* | 547.71  ±75.07 | 309.09  ±32.12 | 218.98  ±17.71 |
