# Supplementary material for: Digital twin for sex-specific identification of class III antiarrhythmic drugs based on in vitro measurements, computer models, and machine learning tools
Source: PLoS Comput Biol. 2025 Jul 3;21(7):e1013154. doi: 10.1371/journal.pcbi.1013154 (PMC12510667; doi:10.1371/journal.pcbi.1013154)
Supplement: S11 Text — (DOCX) [file pcbi.1013154.s011.docx]

# S11_Text: Values of biomarkers of the AAD population (virtual male vs. female).

**Table A.** Values of biomarkers of the AAD population (virtual male vs. female). Statistical significance: *p<0.05; **p<0.01; and ***p<0.001.

|  | **Gender** | **RMP**  **(mV)** | **dV/dt_max_**  **(V/s)** | **APA**  **(mV)** | **APD_20_**  **（ms）** | **APD_40_**  **（ms）** | **APD_50_**  **（ms）** | **APD_90_**  **（ms）** | **APD_tri_**  **（ms）** | **CTA**  **(nM)** | **CT_max_**  **(nM)** | **CTD_50_**  **(ms)** | **CTD_90_**  **(ms)** | **CTD_tri_**  **(ms)** | **CTD**  **(nM)** |
| --- | --- | --- | --- | --- | --- | --- | --- | --- | --- | --- | --- | --- | --- | --- | --- |
| **Class Ⅲ** | Male (*n*=15,221) | -76.31  ±1.18 | 192.53  ±34.37 | 115.45  ±3.39 | 6.09  ±3.96 | 84.13  ±16.95 | 123.06  ±15.18 | 284.11  ±21.44 | 161.05  ±14.70 | 249.17  ±93.22 | 465.92  ±99.80 | 242.40  ±44.87 | 551.44  ±74.84 | 309.04  ±32.30 | 219.56  ±18.02 |
|  | Female  (*n*=16,621) | -75.55  ±1.09*** | 181.25  ±30.35*** | 114.94  ±3.33*** | 6.40  ±4.98*** | 99.23  ±19.69*** | 140.29  ±18.45*** | 327.34  ±32.76*** | 187.05  ±21.45*** | 294.76  ±101.83*** | 515.02  ±107.14*** | 236.99  ±45.84*** | 544.09  ±74.22*** | 307.10  ±31.07*** | 223.45  ±17.18*** |
| **non-Class Ⅲ** | Male  (*n*=13,746) | -76.44  ±1.31 | 189.66  ±40.40 | 114.26  ±3.97 | 5.78  ±0.74 | 77.003  ±16.25 | 117.29  ±13.26 | 275.47  ±18.46 | 158.18  ±14.94 | 239.80  ±90.421 | 454.42  ±98.08 | 241.59  ±44.53 | 552.74  ±74.74 | 311.15  ±32.31 | 217.37  ±17.93 |
|  | Female  (*n*=15,011) | -75.42  ±1.19*** | 170.59  ±34.25*** | 113.05  ±3.96*** | 6.16  ±0.85*** | 92.47  ±17.64*** | 133.93  ±13.76*** | 314.49  ±17.22*** | 180.56  ±15.04*** | 278.24  ±99.97*** | 495.79  ±107.05*** | 235.77  ±45.40*** | 542.89  ±75.07*** | 307.12  ±31.83*** | 220.51  ±17.36*** |
| **All** | Male  (*n*=28,967) | -76.38±  1.25 | 191.07±  37.57 | 114.85±  3.74 | 5.93±  0.87 | 80.52±  16.98 | 120.13±  14.52 | 279.73±  20.45 | 159.60±  14.89 | 244.42±  91.93 | 460.09±  99.10 | 241.99±  44.70 | 552.10±  74.79 | 310.11±  32.32 | 218.45±  18.01 |
|  | Female  (*n*=31,632) | -75.48±  1.15*** | 175.44±  32.96*** | 113.91±  3.81*** | 6.27±  0.92*** | 95.55±  18.90*** | 136.83±  16.37*** | 320.34±  26.29*** | 183.51±  18.53*** | 285.76±  101.15*** | 504.55±  107.51*** | 236.33±  45.61*** | 543.43±  74.69*** | 307.11±  31.48*** | 221.85±  17.34*** |
