# Supplementary material for: Digital twin for sex-specific identification of class III antiarrhythmic drugs based on in vitro measurements, computer models, and machine learning tools
Source: PLoS Comput Biol. 2025 Jul 3;21(7):e1013154. doi: 10.1371/journal.pcbi.1013154 (PMC12510667; doi:10.1371/journal.pcbi.1013154)
Supplement: S12 Text — (DOCX) [file pcbi.1013154.s012.docx]

# S12_Text: The illustration of biomarkers extracted for each sample in the populations.


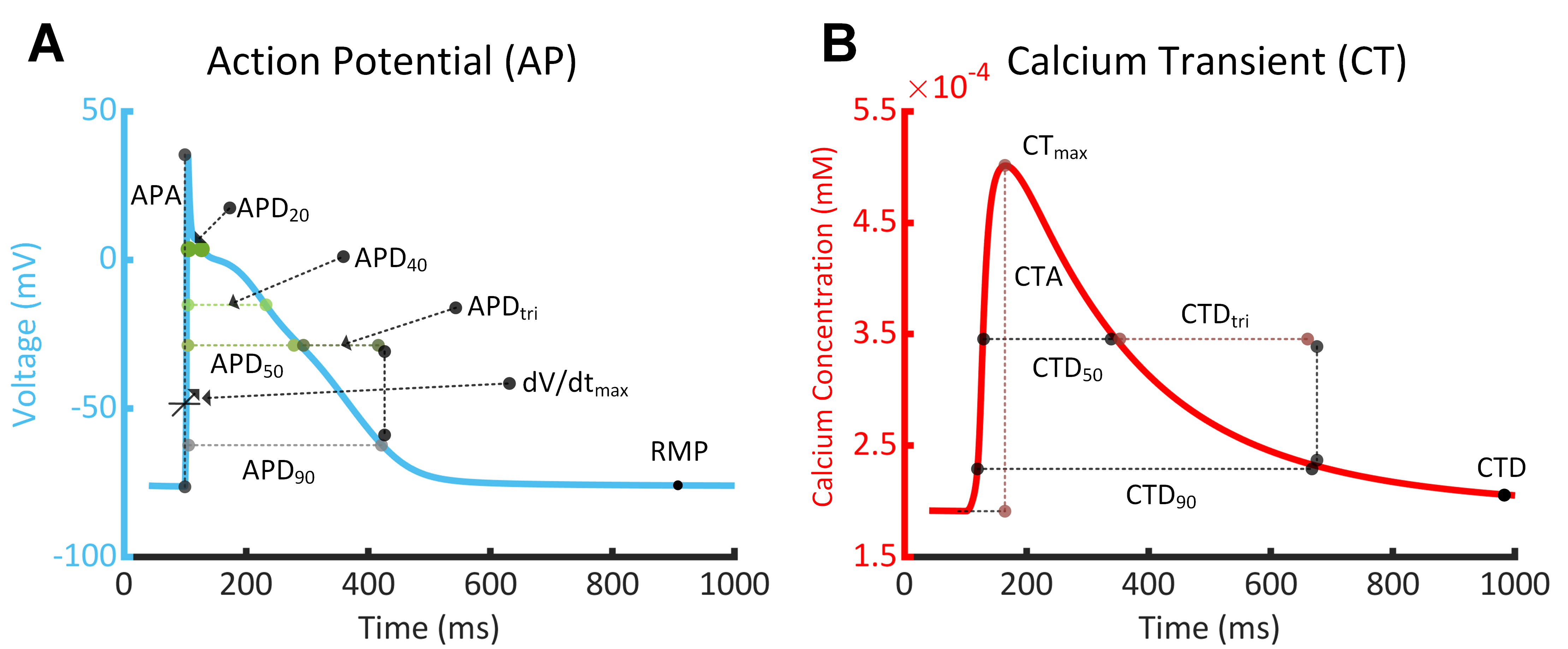


**Fig A.** The illustration of biomarkers extracted for each sample in the populations. (A) biomarkers extracted from the AP recording consisted of RMP, dV/dt_max_, APA, APD_20_, APD_40_, APD_50,_ APD_90_ and APD_tri_; (B) biomarkers extracted from the CT recording consisted of CTD, CT_max_, CTA, CTD_50_, CTD_90_ and CTD_tri_.
