# Supplementary material for: Digital twin for sex-specific identification of class III antiarrhythmic drugs based on in vitro measurements, computer models, and machine learning tools
Source: PLoS Comput Biol. 2025 Jul 3;21(7):e1013154. doi: 10.1371/journal.pcbi.1013154 (PMC12510667; doi:10.1371/journal.pcbi.1013154)
Supplement: S13 Text — (DOCX) [file pcbi.1013154.s013.docx]

# S13_Text: Description of machine learning algorithms.

**Table A.** Hyperparameters which were optimized for ML model.

| Model | Hyperparameters | Values of ML model |
| --- | --- | --- |
| LR | 'C': {0.001,0.01,0.1,1,10,100} | C: 100 |
| SVM | 'kernel': {'rbf', 'linear'}  'C': {0.1,1,10,100},  'gamma': {1,0.1,0.01,0.001} | 'kernel': 'rbf'  'C': 100,  'gamma': 0.1, |
| NB | NAN | NAN |
| XGB | 'n_estimators': {50, 100, 200},  'learning_rate': {0.01, 0.1, 0.3},  'max_depth': {3, 5, 7}, | 'n_estimators': 200  'learning_rate': 0.01,  'max_depth': 5, |
| RF | 'criterion': {'entropy', 'gini'}  'max depth’: {100,200},  'max_features': {2,3},  'min_samples_leaf': {3, 4, 5},  'min_samples_split': ^1^,  'n_estimators': {100, 200}, | 'criterion': 'entropy',  'max_depth': 100,  'max_features': 3,  'min_samples_leaf': 5,  'min_samples_split': 8,  'n_estimators': 200 |
| KNN | 'n_neighbors': {3,5,11,19} | 'n_neighbors': 19 |
