# Supplementary material for: Digital twin for sex-specific identification of class III antiarrhythmic drugs based on in vitro measurements, computer models, and machine learning tools
Source: PLoS Comput Biol. 2025 Jul 3;21(7):e1013154. doi: 10.1371/journal.pcbi.1013154 (PMC12510667; doi:10.1371/journal.pcbi.1013154)
Supplement: Attachment — Submitted filename: Respond.pdf [file pcbi.1013154.s016.pdf]

Professor Yang Lu, Ph.D., Academic Editor
Professor Jason Haugh, Section Editor
PLOS Computational Biology

Dear Editor-in-Chief,

I am writing to submit our revised MS entitled "*A Novel Pipeline for Identifying Gender-Specific Class III Antiarrhythmic Drugs Based on In Vitro Measurements, In-Silico Models and Machine Learning Tools*" (Manuscript ID PCOMPBIOL-D-24-01299) for consideration for publication in PLOS Computational Biology.

We thank all Reviewers and editors for their overall positive evaluation of our study and helpful criticisms and suggestions. We believe the revisions made in all three main sections (structure, description, and discussion) as per the editor's and reviewers' suggestions have significantly improved our manuscript and clarified our data presentation. To expedite the review, we present our revisions to the manuscript in blue font. We have made the following significant changes to improve the paper:

1. Regarding concerns of Reviewer #2 about data generation and trustworthiness, we have provided a detailed and comprehensive account of the screening process and the relevant standers (i.e., ASME V&V 40 standard and the comprehensive in vitro proarrhythmia assay (CiPA)). In the revised MS, we updated the method section (***Pages 20-22, lines 599-641***), added a new Figure (***Supplementary Figure S2***) and a new Table (***Supplementary Table S)***
2. Regarding concerns of Reviewer #2 and Reviewer #3 about feature correlation and analysis, we have conducted the principal component analysis and ablation experiments to strengthen our claim that "The main difference between Male and Female classifiers was in the ranking of ΔRMP and ΔAPA." In the revised MS, we updated the result part (***Pages 11-13, lines 278-331***), and provided three new tables (***Table 4, Table 5 and Table 6***).
3. Regarding concerns of Reviewer #3 and Reviewer #1 about the contribution/innovation of methodology, we have emphasized that the focus of this paper is on the impact of gender factors on drug efficacy and the underlying mechanisms using an existing method. In the revised MS, we have provided several new sections (*author summary* ***(Page 3, Lines 40-57)***, *main contributions* ***(Page 13, Lines 343- 356),*** *potential applications in clinical practice* ***(Page 16, Lines 434-461)*** and *Comparison with previous modelling studies* ***(Pages 15-16, Lines 411-432)***) to clarify it.
4. Regarding concerns of Reviewer #3 about data and code availability, we have provided the dataset(https://drive.google.com/drive/folders/1Novsn7TbeEuLiNKdqsuUEPcoXsacV3La?usp=drive_link) and related codes (https://github.com/xuanyuanzaishui/Sex-Specific-Classification-of-Antiarrhythmic-Drugs).
5. Regarding concerns of Reviewer #1 about class imbalance and metric suitability, we have considered Matthews correlation coefficient (MCC) that is less affected by class imbalance. In the revised MS, we have provided updated results ***(Pages 22-23, Lines 666- 675)*** and five

tables (***Table 1, 2, 3, 4 and 5***).

We hope that our detailed response and text revision are sufficient for acceptance of our MS in PLOS Computational Biology.

Thank you very much for your consideration,

Sincerely,

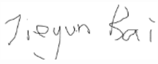

Jieyun Bai, Ph.D.,

Auckland Bioengineering Institute,

The University of Auckland, Auckland, New Zealand

Tel: +64 022 090 0945; Email: bai_jieyun@126.com

Webpage: https://profiles.auckland.ac.nz/jbai996

Reviewer #1:

Summary: The authors propose an ensemble of ML models that incorporate gender to predict the label of a drug (Class III or non-Class III).

1. **Clarity:** The paper is generally written. However, I'm not sure if I understand what the machine learning models are trying to predict. Does it try to predict whether a compound is a class III AAD for a given person (different people view the drug as a different class, i.e., individualized class), or for all people (all people view the drug as a predicted class, i.e., general class)? Since the authors mention "personalized treatment" in conclusion, I tend to believe the classifier is predicting drug class for each person. But for the task, it makes more sense to predict general class. If it is to predict individualized class, why would an individual-based classification of a certain drug be useful? Looking at the person's detailed physiology and drug mechanism is more useful than a coarse class. If it is to predict general class, how is it linked to personalized treatment?

   A new section is suggested to formulate the problem, clarifying what the inputs, and outputs, are.

Respond: Thank you for your valuable comments. Our machine learning models are designed to strike a balance between general drug classification and gender considerations. They provide a powerful tool for drug screening and can contribute to 'personalized' treatment by taking into account gender differences. In the revised MS, we have added a new section "*Author Summary*" to formulate the problem, clarifying what the inputs, and outputs are.

In terms of predicting for all people (general class), our models aim to classify drugs into Class III AADs or non-Class III AADs based on a comprehensive analysis of biomarkers and electrophysiological responses.

However, when we mention 'personalized' (or sex-special) treatment, it does not mean that our classifier is strictly predicting a drug class for each individual. Instead, the individualized aspect comes into play through the consideration of gender differences. We have shown that male and female patients respond differently to drugs, and by developing sex-specific classifiers, we are able to take a step towards personalized medicine.

Changes in Text (Page 3, Lines 40-57)

Author Summary

The challenges posed by the cross-class characteristics of drugs and gender differences in drug response are significant in modern medicine. Traditional methods of drug classification fall short when dealing with drugs that act on multiple targets across different classes. Additionally, male and female patients often respond differently to the same drug due to variations in ion currents, hormonal influences, and calcium handling properties. This necessitates a shift in approach to drug classification and prescribing. In this study, we introduce an innovative pipeline that combines in vitro measurements, in silico models, and machine learning tools to address these challenges. We create a population of calibrated sex-specific models to simulate the effects of drugs on action potential (AP) and calcium transient (CT). By extracting biomarkers from these simulations, we train machine learning classifiers to predict true Class III antiarrhythmic drugs. Our findings show that sex-specific classifiers significantly outperform non-sex-specific classifiers in predicting drug efficacy. We identify key biomarkers that are important for differentiating male

and female responses to antiarrhythmic drugs (AADs). Our study highlights the potential of computational models and machine learning in enhancing drug screening processes and developing sex-special treatment strategies. Overall, our work provides new insights into the role of gender factors in drug evaluation and paves the way for more effective and sex-special medical care.

2. **Significance:** I'm not sure if I get the importance of the work. For either predicting the individualized, or general class, the pharmacist/doctor still need to look at the actual mode of action to determine the use of a drug to a certain patient. The classfication is more for education purpose. If the author believe otherwise, please clarify.

Respond: Thank you for your valuable comment. We understand your concern regarding the significance of our work. We believe that our research has much more importance than just an educational purpose. It has practical applications in clinical practice, drug development, and understanding disease mechanisms. It provides a valuable tool for pharmacists and doctors to make more informed decisions and offers new insights into the complex field of drug response and personalized medicine. In the revised MS, we have added a new section "The potential applications in clinical practice" to the discussion part.

Changes in Text (Page 16, Lines 434-461)

This study has practical applications in clinical practice, drug development, and understanding disease mechanisms. By providing a more comprehensive understanding of gender differences in drug response, it can lead to more personalized and effective medical care.

In clinical practice, the ability to accurately predict a drug's efficacy and potential adverse reactions is crucial for prescribing the right medication to each patient[63,64]. The sex-specific classifiers developed in this study can provide valuable insights that go beyond simply looking at the drug's mode of action. By considering gender differences, clinicians can make more informed decisions when prescribing antiarrhythmic drugs. For example, knowing that female patients are more likely to experience certain adverse drug reactions due to differences in ion currents and calcium handling properties[65], doctors can adjust the dosage or choose a different drug altogether. This approach can lead to better patient outcomes, reduced risk of adverse events, and more effective medical care.

The pipeline developed in this study can enhance the drug development and screening process. By accurately classifying drugs based on their effects on different patient populations, researchers can identify potential candidates for further development more efficiently[66]. Additionally, understanding the gender-specific differences in drug response can help in tailoring drug formulations and clinical trials to better meet the needs of different patient groups. This can lead to the development of more targeted and effective drugs, reducing the time and cost of drug development.

Studying the ionic mechanisms underlying gender differences in drug response can contribute to a better understanding of the pathophysiology of atrial fibrillation and other cardiac rhythm disorders[2]. This knowledge can lead to the development of new therapeutic strategies and interventions that take into account gender-specific factors. For example, understanding the role

of estrogen in calcium handling properties can help in developing drugs that target these specific pathways, potentially reducing the risk of atrial arrhythmogenesis[67].

3. **Originality:** The paper is original by incorporating a new factor into the class prediction: gender. However, the methodology is not very innovative: it merely separates the data to train two different models for genders.

Respond: Thank you for your valuable comment. We appreciate your recognition of the originality brought by incorporating gender as a factor in class prediction. Although our approach may seem straightforward at first glance, it is a carefully considered strategy that addresses the complex issue of gender differences in drug response. We believe that our research has the potential to have a significant impact on the field of sex-special medicine and drug development. On the one hand, we conducted in-depth analyses of the key biomarkers and ionic mechanisms underlying these gender differences. This provides a deeper understanding of the physiological basis of drug response and can lead to the development of new therapeutic strategies. On the other hand, our use of in vitro measurements, in silico models, and machine learning tools is a novel combination that offers a comprehensive approach to drug classification. While individual components of our methodology may have been used in previous studies, our integrated pipeline is unique and has the potential to make contributions to the field. In the revised MS, we have added a new section "Comparison with previous modelling studies" to the discussion part.

Changes in Text (Pages 15-16, Lines 411-432)

Our study offers a novel perspective on antiarrhythmic drug classification by introducing gender as a pivotal factor. While there may be some parallels between our methodology and previous studies[63,64], our concentrated focus on gender disparities and our integrated approach confer significant value to our research. This study yields fresh insights into the role of gender in drug evaluation and paves the way for more efficacious medical care.

Admittedly, our approach of segregating the data to train two distinct models for different genders might appear straightforward at first glance. However, it represents a targeted response to the extensively documented gender differences in drug response[61,62]. Our exhaustive analyses of key biomarkers and ionic mechanisms distinguish our work from prior studies[46,65]. By delving into the disparities between male and female classifiers and pinpointing the specific elements contributing to these differences, we offer a more comprehensive understanding of drug efficacy and potential adverse reactions.

Furthermore, our amalgamation of in vitro measurements, in silico models, and machine learning tools constitutes a unique methodology that presents several advantages over traditional approaches[66,67]. Previous investigations might have employed one or two of these techniques, but our integrated pipeline provides a more comprehensive and accurate assessment of drug effects. For instance, in silico models enable us to simulate the impacts of drugs on a diverse population of cardiomyocytes. Simultaneously, machine learning tools assist us in identifying patterns and predicting drug efficacy. In vitro measurements serve as a ground truth for our simulations and aid in validating our results.

4. **Scientific Soundness:** The two class label: Class III and Non-Class III are imbalanced (3,1842 for the former and 28,757 for the latter, respectively). However, the chosen

classification metrics (e.g., accuracy, sensitivity, specificity etc) are skewed under class imbalance. The authors should provide detailed discussion for the impact of class imbalance and incorporate some unbiased metrics to better evaluate the performance. A useful resource: [1]. [1] Luque, Amalia, et al. "The impact of class imbalance in classification performance metrics based on the binary confusion matrix." *Pattern Recognition* 91 (2019): 216-231.

Respond: Thank you for your astute observation. We acknowledge the issue of class imbalance in our dataset and understand the importance of addressing it for a more accurate and comprehensive evaluation of our models. We have referred to the resource you provided, [1] Luque et al. "The impact of class imbalance in classification performance metrics based on the binary confusion matrix," to gain further insights into the issue and to ensure that our evaluation is as robust as possible. In addition, we have considered Matthews correlation coefficient (MCC) that is less affected by class imbalance. This metric can provide a more accurate assessment of our classifiers' performance and help us better understand their effectiveness in handling imbalanced datasets.

Changes in Text (Pages 22-23, Lines 666- 675)

Quantitative assessment was performed by using the following metrics: accuracy (ACC), sensitivity (SEN), specificity (SPE), F1 score (F1), area under the receiver operating characteristic (ROC) curve (AUC) and Mathew Correlation Coefficient (MCC)[73]. They are defined as

$$MCC = (TN * TP - FP * FN)/\sqrt{(TP + FP)(TP + FN)(TN + FP)(TN + FN)}$$

Table 1. *Performance of sex-specific and non-sex-specific classifiers.*

Type	Models	ACC	SEN	SPE	F1	MCC	AUC
Sex-special model (Male)	LR	0.6553 (0.6460,0.6646)	0.6063 (0.5952,0.6174)	0.6198 (0.6046,0.6351)	0.6122 (0.6071,0.6173)	0.3025 (0.2820,0.3229)	0.6507 (0.6428,0.6584)
	SVM	**0.8802 (0.8751,0.8853)**	0.7635 (0.7569,0.7701)	**0.9454 (0.9320,0.9587)**	**0.8450 (0.8389,0.8510)**	**0.7620 (0.7478,0.7762)**	**0.8659 (0.8607,0.8703)**
	NB	0.5993 (0.5949,0.6036)	**0.9346 (0.9266,0.9427)**	0.5166 (0.5137,0.5195)	0.6652 (0.6617,0.6687)	0.3350 (0.3227,0.3474)	0.6422 (0.6380,0.6463)
	XGB	0.8696 (0.8614,0.8777)	0.7826 (0.7707,0.7944)	0.8975 (0.8849,0.9100)	0.8368 (0.8267,0.8469)	0.7129 (0.7000,0.7259)	0.8586 (0.8503,0.8669)
	RF	0.8471 (0.8410,0.8531)	0.7550 (0.7459,0.7641)	0.8702 (0.8563,0.8841)	0.8084 (0.8014,0.8154)	0.6929 (0.6767,0.7091)	0.8354 (0.8296,0.8413)
	KNN	0.8263 (0.8228,0.8297)	0.7010 (0.69430.7077)	0.8663 (0.8554,0.8772)	0.7752 (0.7716,0.7788)	0.6461 (0.6360,0.6561)	0.8103 (0.8073,0.8133)
Sex-special model (Female)	LR	0.6707 (0.6666,0.6747)	0.5790 (0.5760,0.5820)	0.6225 (0.6166,0.6284)	0.5999 (0.5961,0.6038)	0.3210 (0.3108,0.3312)	0.6589 (0.6551,0.6627)
	SVM	**0.8774 (0.8738,0.8809)**	0.7660 (0.7613,0.7707)	**0.9484 (0.9442,0.9525)**	**0.8475 (0.8429,0.8520)**	**0.7601 (0.7509,0.7691)**	**0.8663 (0.8626,0.8699)**
	NB	0.5877 (0.5833,0.5921)	**0.9183 (0.9135,0.9232)**	0.5207 (0.5177,0.5237)	0.6647 (0.6627,0.6666)	0.2918 (0.2839,0.2996)	0.6206 (0.6170,0.6242)
	XGB	0.8579 (0.8540,0.8619)	0.7788 (0.7726,0.7850)	0.8894 (0.8823,0.8965)	0.8295 (0.8249,0.8342)	0.6896 (0.6765,0.7026)	0.8499 (0.8460,0.8538)
	RF	0.8320 (0.8292,0.8347)	0.7476 (0.7421,0.7531)	0.8571 (0.8521,0.8620)	0.7980 (0.7946,0.8014)	0.6720 (0.6617,0.6823)	0.8235 (0.8206,0.8263)
	KNN	0.8201 (0.8159,0.8242)	0.7008 (0.6951,0.7065)	0.8704 (0.8621,0.8787)	0.7757 (0.7708,0.7806)	0.6396 (0.6286,0.6507)	0.8081 (0.8040,0.8122)
Non-sex-special model	LR	0.6717 (0.6696,0.6737)	0.8385 (0.8342,0.8427)	0.6501 (0.6478,0.6524)	0.7323 (0.7309,0.7338)	0.3430 (0.3389,0.3471)	0.6589 (0.6566,0.6611)
	SVM	**0.8095 (0.8064,0.8127)**	0.8253 (0.8187,0.8319)	**0.8217 (0.8177,0.8257)**	**0.8226 (0.8193,0.8259)**	**0.6179 (0.6101,0.6258)**	**0.8085 (0.8053,0.8117)**
	NB	0.6325 (0.6279,0.6371)	**0.9012 (0.8958,0.9066)**	0.6055 (0.6025,0.6084)	0.7243 (0.7208,0.7278)	0.2776 (0.2660,0.2892)	0.6118 (0.6072,0.6165)
	XGB	0.7914 (0.7879,0.7949)	0.7959 (0.7868,0.8050)	0.8112 (0.8079,0.8145)	0.8034 (0.7991,0.8077)	0.5815 (0.5748,0.5881)	0.7910 (0.7878,0.7943)
	RF	0.7650 (0.7615,0.7685)	0.7858 (0.7793,0.7923)	0.7778 (0.7744,0.7813)	0.7818 (0.7781,0.7854)	0.5264 (0.5220,0.5309)	0.7634 (0.7600,0.7668)
	KNN	0.7844 (0.7801,0.7886)	0.8014 (0.7959,0.8068)	0.7972 (0.7922,0.8023)	0.7993 (0.7954,0.8032)	0.5665 (0.5578,0.5751)	0.7831 (0.7787,0.7874)

Table 2. *Performance of sex-biased classifiers (i.e., models trained and tested on the data set with virtual patients of the different gender). M-male and F-female.*

Type	Models	ACC	SEN	SPE	F1	MCC	AUC
Male on Female	LR	0.6473 (0.6470,0.6476)	0.5248 (0.5228,0.5268)	0.6018 (0.6012,0.6024)	0.5607 (0.5596,0.5617)	0.2702 (0.2694,0.2709)	0.6320 (0.6316,0.6325)

	SVM	0.8472 (0.8465,0.8479)	0.7601 (0.7580,0.7622)	**0.8671 (0.8665,0.8678)**	0.8101 (0.8090,0.8112)	0.6874 (0.6860,0.6889)	0.8363 (0.8355,0.8372)
	NB	0.5808 (0.5800,0.5817)	**0.9227 (0.9197,0.9256)**	0.5061 (0.5056,0.5067)	0.6537 (0.6534,0.6540)	0.2959 (0.2950,0.2967)	0.6234 (0.6230,0.6238)
	XGB	**0.8492 (0.8481,0.8502)**	0.7997 (0.7963,0.8031)	0.8408 (0.8375,0.8441)	**0.8197 (0.8186,0.8209)**	**0.6909 (0.6889,0.6930)**	**0.8430 (0.8420,0.8440)**
	RF	0.8396 (0.8389,0.8402)	0.7775 (0.7741,0.7809)	0.8368 (0.8357,0.8379)	0.8060 (0.8047,0.8074)	0.6709 (0.6695,0.6723)	0.8318 (0.8308,0.8328)
	KNN	0.8005 (0.7988,0.8021)	0.7166 (0.7143,0.7190)	0.7976 (0.7927,0.8025)	0.7549 (0.7539,0.7560)	0.5900 (0.5865,0.5934)	0.7900 (0.7888,0.7912)
**Female on Male**	LR	0.6446 (0.6430,0.6462)	0.5872 (0.5823,0.5921)	0.6079 (0.6055,0.6104)	0.5974 (0.5951,0.5997)	0.2797 (0.2765,0.2827)	0.6393 (0.6377,0.6408)
	SVM	**0.8695 (0.8685,0.8704)**	0.7427 (0.7394,0.7459)	**0.9545 (0.9530,0.9560)**	0.8353 (0.8337,0.8369)	**0.7458 (0.7442,0.7475)**	**0.8571 (0.8559,0.8582)**
	NB	0.5958 (0.5950,0.5966)	**0.8801 (0.8778,0.8824)**	0.5301 (0.5295,0.5306)	0.6617 (0.6609,0.6624)	0.2790 (0.2771,0.2809)	0.6221 (0.6213,0.6229)
	XGB	0.8638 (0.8628,0.8649)	0.7882 (0.7853,0.7910)	0.8939 (0.8896,0.8983)	**0.8377 (0.8368,0.8386)**	0.7256 (0.7231,0.7280)	0.8564 (0.8556,0.8573)
	RF	0.8530 (0.8522,0.8537)	0.7458 (0.7437,0.7479)	0.9106 (0.9077,0.9135)	0.8200 (0.8191,0.8209)	0.7078 (0.7059,0.7096)	0.8430 (0.8423,0.8438)
	KNN	0.8206 (0.8197,0.8215)	0.6936 (0.6898,0.6973)	0.8817 (0.8765,0.8870)	0.7764 (0.7756,0.7772)	0.6429 (0.6404,0.6453)	0.8089 (0.8082,0.8095)

Table 3. *Performance of SVM using different numbers of top biomarkers based on feature importance ranking shown in Figure 9.*

Type	Number	ACC	SEN	SPE	F1	MCC	AUC
**Sex-special model (Male)**	3	0.7519 (0.7461,0.7577)	0.6406 (0.6306,0.6506)	0.7424 (0.7345,0.7504)	0.6877 (0.6798,0.6957)	0.4876 (0.4753,0.4998)	0.7377 (0.7315,0.7438)
	5	0.8021 (0.7964,0.8079)	0.6781 (0.6677,0.6885)	0.8269 (0.8177,0.8362)	0.7451 (0.7371,0.7530)	0.5938 (0.5817,0.6060)	0.7862 (0.7802,0.7923)
	7	0.8590 (0.8543,0.8637)	0.7285 (0.7179,0.7391)	0.9250 (0.9192,0.9309)	0.8150 (0.8079,0.8221)	0.7169 (0.7075,0.7263)	0.8423 (0.8369,0.8476)
	9	0.8694 (0.8612,0.8776)	0.7446 (0.7339,0.7552)	0.9364 (0.9198,0.9530)	0.8294 (0.8191,0.8398)	0.7385 (0.7206,0.7565)	0.8534 (0.8452,0.8616)
	11	0.8738 (0.8674,0.8802)	0.7530 (0.7414,0.7646)	0.9391 (0.9265,0.9517)	0.8357 (0.8272,0.8443)	0.7472 (0.7336,0.7608)	0.8583 (0.8516,0.8650)
	**14**	**0.8802 (0.8751,0.8853)**	**0.7635 (0.7569,0.7701)**	**0.9454 (0.9320,0.9587)**	**0.8450 (0.8389,0.8510)**	**0.7620 (0.7478,0.7762)**	**0.8655 (0.8607,0.8703)**
**Sex-special model (Female)**	3	0.7299 (0.7254,0.7344)	0.6576 (0.6513,0.6638)	0.7129 (0.7066,0.7192)	0.6841 (0.6789,0.6893)	0.4501 (0.4409,0.4592)	0.7227 (0.7182,0.7272)
	5	0.7843 (0.7783,0.7902)	0.7116 (0.7027,0.7204)	0.7834 (0.7771,0.7898)	0.7458 (0.7382,0.7533)	0.5612 (0.5490,0.5735)	0.7770 (0.7708,0.7833)
	7	0.8511 (0.8464,0.8558)	0.7395 (0.7301,0.7489)	0.9088 (0.9020,0.9155)	0.8154 (0.8090,0.8219)	0.7033 (0.6938,0.7128)	0.8400 (0.8350,0.8450)
	9	0.8635 (0.8578,0.8692)	0.7460 (0.7388,0.7532)	0.9338 (0.9231,0.9446)	0.8294 (0.8224,0.8363)	0.7306 (0.7184,0.7429)	0.8518 (0.8461,0.8575)
	11	0.8703 (0.8656,0.8750)	0.7569 (0.7492,0.7646)	0.9398 (0.9332,0.9463)	0.8385 (0.8323,0.8446)	0.7443 (0.7346,0.7539)	0.8590 (0.8541,0.8639)
	**14**	**0.8774 (0.8738,0.8809)**	**0.7660 (0.7613,0.7707)**	**0.9484 (0.9442,0.9525)**	**0.8475 (0.8429,0.8520)**	**0.7601 (0.7509,0.7691)**	**0.8663 (0.8626,0.8699)**
**Non-sex-special model**	3	0.7337 (0.7269,0.7404)	0.8072 (0.8020,0.8124)	0.7262 (0.7196,0.7328)	0.7646 (0.7590,0.7701)	0.4634 (0.4497,0.4771)	0.7280 (0.7210,0.7349)
	5	0.7512 (0.7453,0.7571)	0.8115 (0.8034,0.8195)	0.7463 (0.7417,0.7508)	0.7775 (0.7718,0.7832)	0.4988 (0.4867,0.5109)	0.7466 (0.7407,0.7524)
	7	0.7891 (0.7853,0.7930)	0.8097 (0.8033,0.8161)	0.7993 (0.7946,0.8041)	0.8045 (0.8007,0.8082)	0.5759 (0.5680,0.5837)	0.7876 (0.7837,0.7915)
	9	0.8029 (0.7993,0.8066)	0.8161 (0.8108,0.8215)	0.8161 (0.8100,0.8222)	0.8161 (0.8130,0.8192)	0.6040 (0.5964,0.6115)	0.8019 (0.7980,0.8058)
	11	0.8041 (0.8025,0.8058)	0.8163 (0.8078,0.8248)	0.8178 (0.8140,0.8217)	0.8170 (0.8144,0.8197)	0.6065 (0.6034,0.6095)	0.8032 (0.8019,0.8045)
	**14**	**0.8095 (0.8064,0.8127)**	**0.8253 (0.8187,0.8319)**	**0.8217 (0.8177,0.8257)**	**0.8226 (0.8193,0.8259)**	**0.6179 (0.6101,0.6258)**	**0.8085 (0.8053,0.8117)**

5. Figure2A, 3A, 4A: The color lines are overlapped and it is hard to see if they're distributed similarly or differently. Maybe think of adjusting the transparency of the lines so the overlaid parts can be seen clearly. Also, the captions say "representative traces" how do the authors ensure the selected lines are representative?

Respond: Thank you for your astute comments. We are grateful for your careful scrutiny of the figures in our paper. Concerning the issue of overlapping color lines in Figures 2A, 3A, and 4A, we wholeheartedly agree that it poses challenges in discerning whether the lines are distributed similarly or differently. We have taken your suggestion seriously and implemented adjustments. Regarding the description of "representative traces," we acknowledge that our initial expression

was inaccurate. In fact, the figures are based on comprehensive data from all samples. We have clarified this in the figure captions and relevant sections of the text to ensure that readers have a clear understanding of the source and nature of the traces presented. Once again, we appreciate your feedback and look forward to presenting an improved version of our work.

Changes in Text (Figures 2-4)

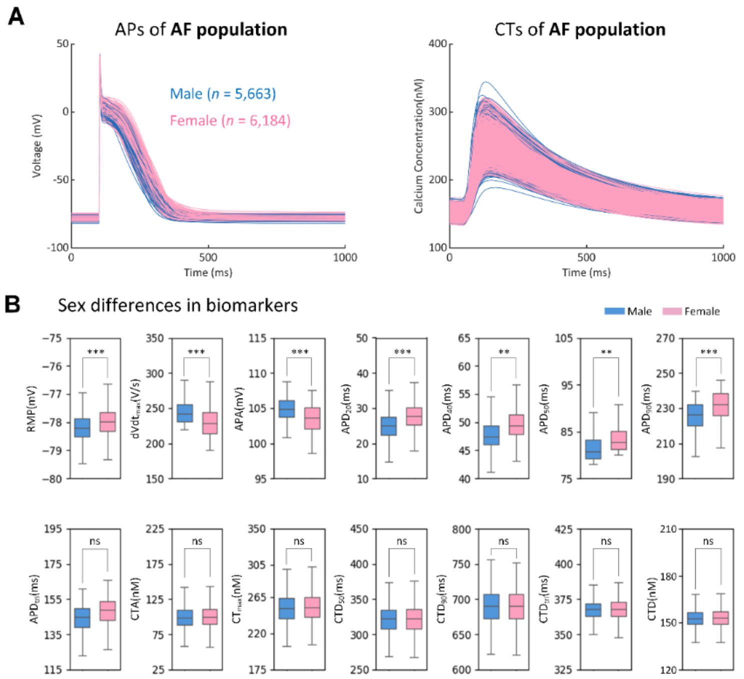

Figure 2. *Sex differences in the AF population of human atrial cell models (5,663 males and 6,184 females). (A) Traces of action potential (AP) and calcium transient (CT). (B) Sex differences in AP biomarkers (RMP, dV/dt_{max}, APA, APD_{20}, APD_{40}, APD_{50}, APD_{90} and APD_{tri}) and CT biomarkers (CTA, CT_{max}, CTD_{50}, CTD_{90}, CTD_{tri} and CTD). The p-values are indicated and the stars *, **, *** indicate a p-value < 0.05, 0.01 and 0.001, respectively. ns indicates not significant.*

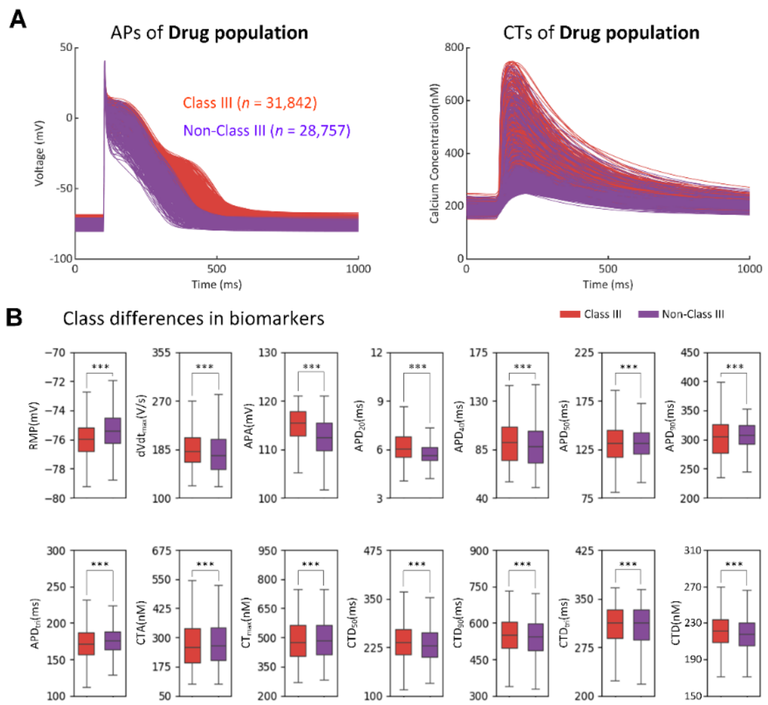

***Figure 3**. Class differences in the drug population of human atrial cell models (31,842 in the Class III and 28,757 in the non-Class III). (A) Traces of action potential (AP) and calcium transient (CT). (B) Class differences in AP biomarkers and CT biomarkers. *** indicates a p-value < 0.001.*

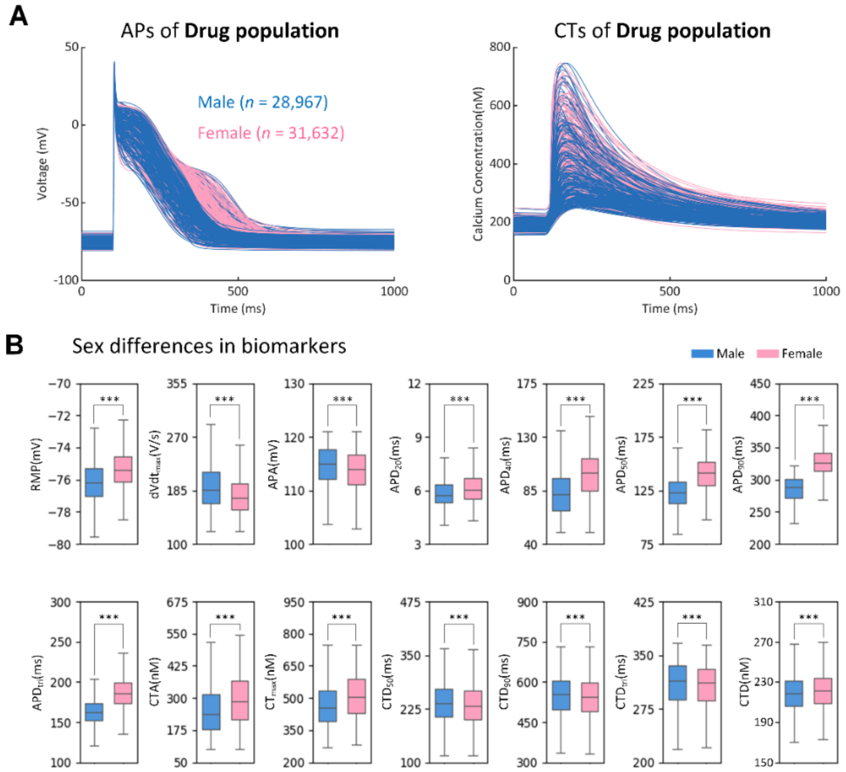

***Figure 4.** Sex differences in the drug population of human atrial cell models (28,967 males and 31,632 females). (A) Traces of action potential (AP) and calcium transient (CT). (B) Sex*

*differences in AP biomarkers and CT biomarkers. *** indicates a p-value < 0.001.*

6. **Conclusion:** The paper has modest innovation in its methodology. The impact and the scientific soundness should be further elucidated. Hence a major revision is granted.

Respond: Thank you for your valuable feedback. We are grateful for the opportunity to revise our manuscript and work hard to address your concerns and strengthen the impact and scientific soundness of our work.

- Acknowledging that our method is an existing method, we emphasize that the focus of this paper is on the impact of gender factors on drug efficacy and the underlying mechanisms. Our aim was not necessarily to introduce a completely novel methodology in terms of the analysis technique itself. Instead, we utilized this existing approach to explore a new aspect of drug response - the role of gender. By integrating in vitro measurements, in silico models, and machine learning tools in a unique way, we were able to specifically address the problem of gender-specific drug response in atrial fibrillation. This combination of approaches provides a more comprehensive understanding of the complex interactions between drugs, gender, and cardiac electrophysiology.
- For the impact of our work, we have expanded on the potential applications and implications of our findings. We have discussed how our research can contribute to personalized medicine by providing clinicians with more accurate tools for prescribing drugs based on gender differences. Additionally, we have explored the broader implications for drug development and screening, highlighting how our approach can lead to the development of more effective and targeted drugs.
- Regarding scientific soundness, we have addressed the issues raised regarding class imbalance and ensure that we use appropriate metrics and techniques to evaluate the performance of our models.

Changes in Text (See respond to Comments #1-4)

The challenges posed by the cross-class characteristics of drugs and gender differences in drug response are significant in modern medicine. Traditional methods of drug classification fall short when dealing with drugs that act on multiple targets across different classes. Additionally, male and female patients often respond differently to the same drug due to variations in ion currents, hormonal influences, and calcium handling properties. This necessitates a shift in approach to drug classification and prescribing. In this study, we introduce a pipeline that combines in vitro measurements, in silico models, and machine learning tools to address these challenges. We create a population of calibrated sex-specific models to simulate the effects of drugs on action potential (AP) and calcium transient (CT). By extracting biomarkers from these simulations, we train machine learning classifiers to predict true Class III antiarrhythmic drugs. Our findings show that sex-specific classifiers significantly outperform non-sex-specific classifiers in predicting drug efficacy. We identify key biomarkers that are important for differentiating male and female responses to antiarrhythmic drugs (AADs). Our study highlights the potential of computational models and machine learning in enhancing drug screening processes and developing sex-special treatment strategies. Overall, our work provides new insights into the role of gender factors in drug evaluation and paves the way for more effective and sex-special medical care.

This study has practical applications in clinical practice, drug development, and understanding disease mechanisms. By providing a more comprehensive understanding of gender differences in

drug response, it can lead to more personalized and effective medical care.

In clinical practice, the ability to accurately predict a drug's efficacy and potential adverse reactions is crucial for prescribing the right medication to each patient[63,64]*. The sex-specific classifiers developed in this study can provide valuable insights that go beyond simply looking at the drug's mode of action. By considering gender differences, clinicians can make more informed decisions when prescribing antiarrhythmic drugs. For example, knowing that female patients are more likely to experience certain adverse drug reactions due to differences in ion currents and calcium handling properties*[65]*, doctors can adjust the dosage or choose a different drug altogether. This approach can lead to better patient outcomes, reduced risk of adverse events, and more effective medical care.*

The pipeline developed in this study can enhance the drug development and screening process. By accurately classifying drugs based on their effects on different patient populations, researchers can identify potential candidates for further development more efficiently[66]*. Additionally, understanding the gender-specific differences in drug response can help in tailoring drug formulations and clinical trials to better meet the needs of different patient groups. This can lead to the development of more targeted and effective drugs, reducing the time and cost of drug development.*

Studying the ionic mechanisms underlying gender differences in drug response can contribute to a better understanding of the pathophysiology of atrial fibrillation and other cardiac rhythm disorders[2]*. This knowledge can lead to the development of new therapeutic strategies and interventions that take into account gender-specific factors. For example, understanding the role of estrogen in calcium handling properties can help in developing drugs that target these specific pathways, potentially reducing the risk of atrial arrhythmogenesis*[67]*.*

quantitative assessment was performed by using the following metrics: accuracy (ACC), sensitivity (SEN), specificity (SPE), F1 score (F1), area under the receiver operating characteristic (ROC) curve (AUC) and Mathew Correlation Coefficient (MCC)[73]*. They are defined as*

$$MCC = (TN * TP - FP * FN)/\sqrt{(TP + FP)(TP + FN)(TN + FP)(TN + FN)}$$

Reviewer #2:

This paper introduces sex-dependent machine learning (ML) algorithms for classifying whether the drug class that a patient with Atrial Fibrillation (AF) has taken belongs to Class III or not, based on action potential (AP) and calcium transient (CT) biomarkers. The results demonstrate that creating two classifiers for different sexes works better compared to a sex-non-specific classifier, and the AP features are more important than the CT features. The authors further tie in the relationship between different ion mechanisms to explain the gender difference in drug response. Overall, the paper is well written with extensive analysis, but the reviewer would still like to point out some issues to be addressed before proceeding with this work to publication.

Major Points

1. First, it seems concerning in section 2.1 that only 11,847 models were accepted from an initial population of 100,000 models. If the simulation only has around a 10% success rate in generating data that aligns with the experimentally determined ranges of biomarkers, it probably will require some further proof to demonstrate that the data generated from the simulation are indeed trustworthy to make the ML results more robust.

Respond: We appreciate your comment and understand your concern regarding the relatively low acceptance rate of models from the initial population. In this study, the model screening process adhered strictly to the ASME V&V 40 standard, which is recognized by the United States Food and Drug Administration (FDA) and offers a risk-based framework for determining the credibility requirements of a computational model. Additionally, it was in accordance with the comprehensive in vitro proarrhythmia assay (CiPA), a novel strategy proposed by expert working groups sponsored by the Cardiac Safety Research Consortium (CSRC), Health and Environmental Sciences Institute (HESI), and the FDA.

In the revised manuscript, a detailed and comprehensive account of the screening process has been provided. As illustrated in Supplementary Figure S2, we initiated with an initial population of 100,000 models. In the first step, 20,963 models with failed repolarization and 5,447 models with abnormal repolarization were excluded, leading to 73,590 models with normal repolarization. Subsequently, the population was further refined by eliminating 61,743 models that did not lie within the range of experimental measurements. The parameters for these experimental measurements and their specific ranges are detailed in Supplementary Table S2. Among the excluded models, 29,700 did not match the characteristics of male atrial fibrillation, and 32,043 did not match those of female atrial fibrillation. Through this meticulous and multi-step screening procedure, we finally obtained 11,847 models, consisting of 5,663 male models and 6,184 female models.

Changes in Text (Pages 20-21, Lines 559- 611)

As illustrated in Supplementary Figure S2, we initiated with an initial population of 100,000 models. In the first step, 20,963 models with failed repolarization and 5,447 models with abnormal repolarization were excluded, leading to 73,590 models with normal repolarization. Subsequently, the population was further refined by eliminating 61,743 models that did not lie within the range of experimental measurements. The parameters for these experimental measurements and their specific ranges are detailed in Supplementary Table S2. Among the excluded models, 29,700 did not match the characteristics of male atrial fibrillation, and 32,043

did not match those of female atrial fibrillation. Through this meticulous and multi-step screening procedure, we finally obtained 11,847 models, consisting of 5,663 male models and 6,184 female models. The distribution of each ion current in males and females is shown in Supplementary Figure S3.

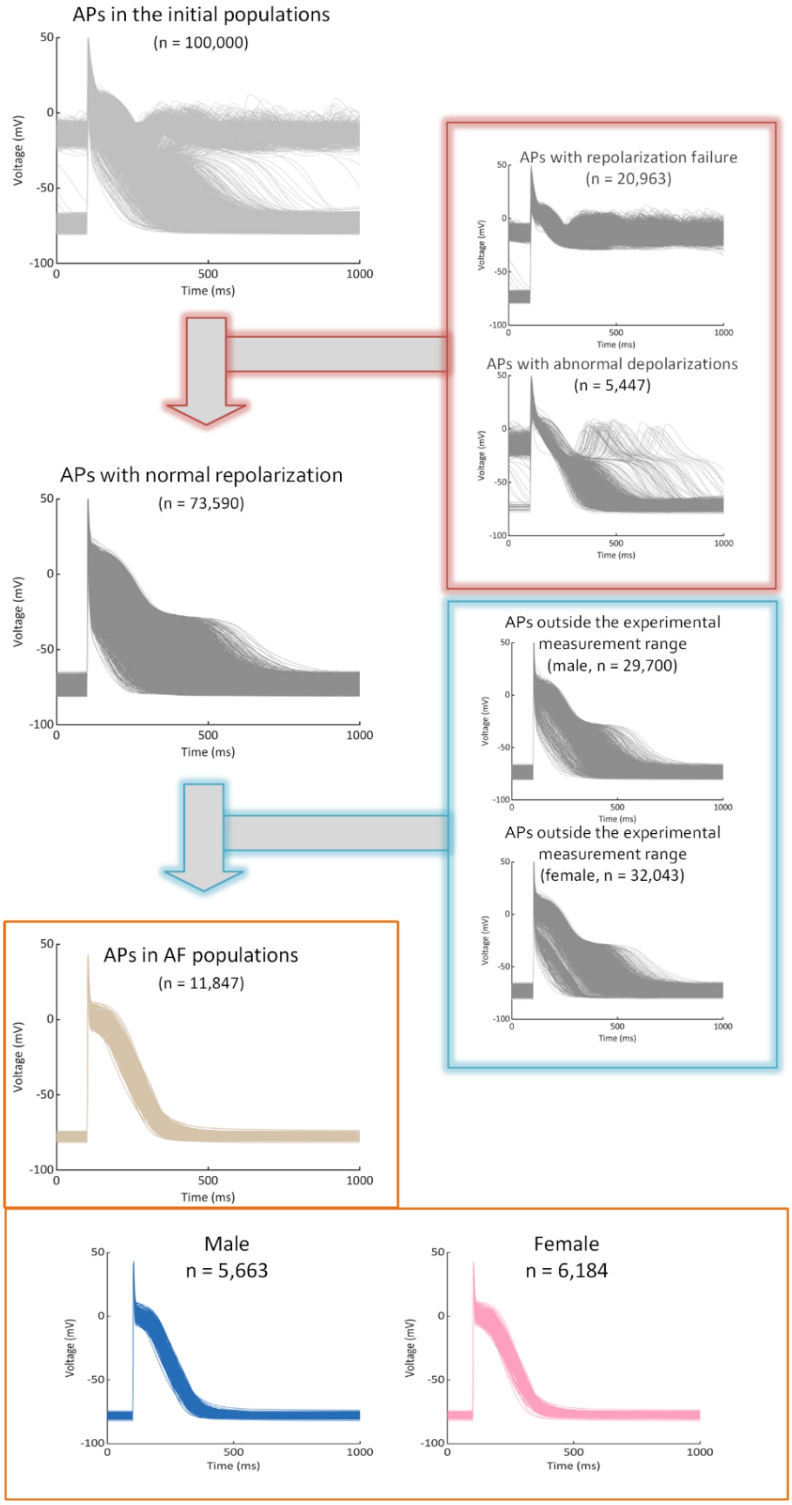

Supplementary Table S2. *The flowchart illustrates the model screening process of constructing AF populations of males and females. AP, action potential.*

Supplementary Table S2. *Values of biomarkers of action potential recordings from*

AF patients (male vs. female).

AP biomarkers	*Male (n=180)*	*Female (n=107)*
RMP (mV)	*-77.57 ± 0.30*	*-77.27 ± 0.39*
dV/dt_{max} (V/S)	*248.23 ± 5.65*	*233.1 ± 7.28*
APA (mV)	*102.20 ± 0.59*	*100.98 ± 0.77*
APD_{20} (ms)	*28.03 ± 1.15*	*29.00 ± 1.48*
APD_{50} (ms)	*99.19 ± 3.09*	*101.79 ± 3.98*
APD_{90}(ms)	*216.01 ± 3.34*	*222.19 ± 4.30*
V20 (mV)	*5.56 ± 0.72*	*6.63 ± 0.93*

2. Similarly, the subsequent generation of drug populations needs some more explanation as well. The only line in the main text about this part of data generation is "By incorporating drug effects on different ion channels, we created an initial population from the AF population and removed models with abnormalities to generate a drug population of 60,599 AP models." It is not clear to the reviewer how the previously sampled 11,847 models can be augmented to 60,599 samples and what abnormalities the authors encountered during the processing procedures.

Respond: Thank you for your comment. We understand the need for more detailed explanation regarding the generation of the drug population.

For each drug, we started with the previously sampled 11,847 models from the atrial fibrillation (AF) population. Take a drug i as an example. The initial population for this drug is also 11,847 models. However, among these models, A_i models with abnormal repolarization are excluded. The number of models included in the drug population for drug is thus 11,847-A_i. In our study, we investigated a total of 12 drugs ($i = 1,2, \ldots 12$). To obtain the final drug population, we summed up the populations for each drug. The final drug population is calculated as (11,847x12-($A_1 + A_2 + \cdots + A_{12}$)). The details for each drug, including the initial population, the number of excluded models, and the final population, are presented in new Supplementary Table S6.

In the revised MS, we've added content on building drug models and screening models.

***Changes in Text* (Pages 21-22, Lines 613- 641)**

To investigate different effects of AADs, the population of sampled models of human atrial electrophysiology for Class III and non-Class III AADs was generated based on models in the AF population. Class III AADs include Amiodarone, Dofetilide, Dronedarone, Ibutilide, Sotalol, and Vernakalant, while non-Class III AADs include Flecainide, Propafenone, Quinidine, Ranolazine, Disopyramide, and Digoxin [11]. A simple pore channel equation was used for drug implementation for each drug that was modeled according to experimental IC50 values to calculate the block of the channel (Supplementary Table S3)41,70. The model used is described as follows:

$$g_{i,drug} = g_i \left[1 + \left(\frac{D}{IC_{50,i}}\right)^h\right]^{-1}$$

where $g_{i,drug}$ is the maximal conductance of channel i in the presence of the drug, g_i is the initial conductance of the channel i for each of the profiles in the AF population of modes, D is the concentration of the drug and $IC_{50,i}$ is the concentration of the drug that reduces by 50% the channel current.

Supplementary Table S3. The list of antiarrhythmic drugs with their concentration(µM), IC50 (µM)

and hill coefficient [2,3].

	Drugs	Concentration (μM)	IC 50 values (hill coefficient)									
			INa	INaL	Ito	ICaL	IKur	IKr	IKs	IK1	INaCa	INaK
**Class III**	Amiodarone	2.0	5 (1.0)	-	3.8 (0.4)	1.5 (0.6)	-	3.0 (1.0)	100 (1.0)	-	3.4 (1.0)	-
	Dofetilide	0.002	0.3805 (0.9)	753.16 (0.3)	0.0188 (0.8)	0.263 (1.3)	-	0.0049 (0.9)	-	0.3943 (0.8)	-	-
	Dronedarone	0.205	0.54 (2.03)	-	-	0.83 (2.75)	1.0 (1.0)	0.0591 (0.8)	5.6 (0.51)	-	-	-
	Ibutilide	0.015	-	-	-	-	-	0.02 (1.0)	-	-	-	-
	Sotalol	14.69	1140000 (0.5)	-	43143.55 (0.7)	7061.527 (0.9)	-	110.6 (0.8)	4221.856 (1.2)	3050.26 (1.2)	-	-
	Vernakalant	30	90 (1.0)	-	15 (1.0)	84 (1.0)	15 (1.0)	20 (1.0)	-	-	-	-
**Non-Class III**	Digoxin	0.04	-	-	-	-	-	0.054 (1.0)	-	-	-	0.12 (1.0)
	Disopyramide	0.742	168.4 (1.0)	-	-	1036.7 (1.0)	-	14.4 (1)	-	-	-	-
	Flecainide	2.5	6.5 (1.0)	-	10.0 (0.8)	27.1 (1.0)	2.9 (1.0)	1.6 (1.0)	-	-	-	-
	Propafenone	0.8	2.5 (1.0)	-	7.2 (1.0)	1.55 (1.0)	-	0.44 (1.0)	16 (1.0)	-	-	-
	Quinidine	3.237	12.329 (1.5)	9.417 (1.3)	3.4874 (1.3)	51.5923 (0.6)	-	0.992 (0.8)	4.8989 (1.4)	39589.919 (0.4)		
	Ranolazine	10	200 (1.0)	-	-	250 (1.0)	-	12 (1.0)	100 (1.0)	-	91 (1.0)	-

All AP models considering AAD effects in the AF population were paced at 1 Hz, and APs were analyzed following a train of 100 periodic stimuli to quantify AP and CT for each model in the Class III and non-Class III AAD subpopulations. Take a drug i as an example. The initial population for this drug is also 11,847 models. However, among these models, A_i models with abnormal repolarization are excluded. The number of models included in the drug population for drug is thus 11,847-A_i. In our study, we investigated a total of 12 drugs ($i = 1,2, \ldots 12$). To obtain the final drug population, we summed up the populations for each drug. The final drug population is calculated as (11,847x12-($A_1 + A_2 + \cdots + A_{12}$)). Finally, 60,599 AP models were generated to form the ADD population, containing 31,842 AP models (15,221 male and 16,621 female AP models) in the Class III AAD subpopulation (Supplementary Figure S3A) and 28,757 AP models (13,746 male and 15,011 female AP models) in the non-Class III subpopulation (Supplementary Figure S3B). The proportion of AP models for each AAD ranged from 7% to 24%. Values of biomarkers of the AAD population can be found in Supplementary Table S4 for Class III vs. non-Class III and in Supplementary Table S5 for virtual male vs. female, respectively.

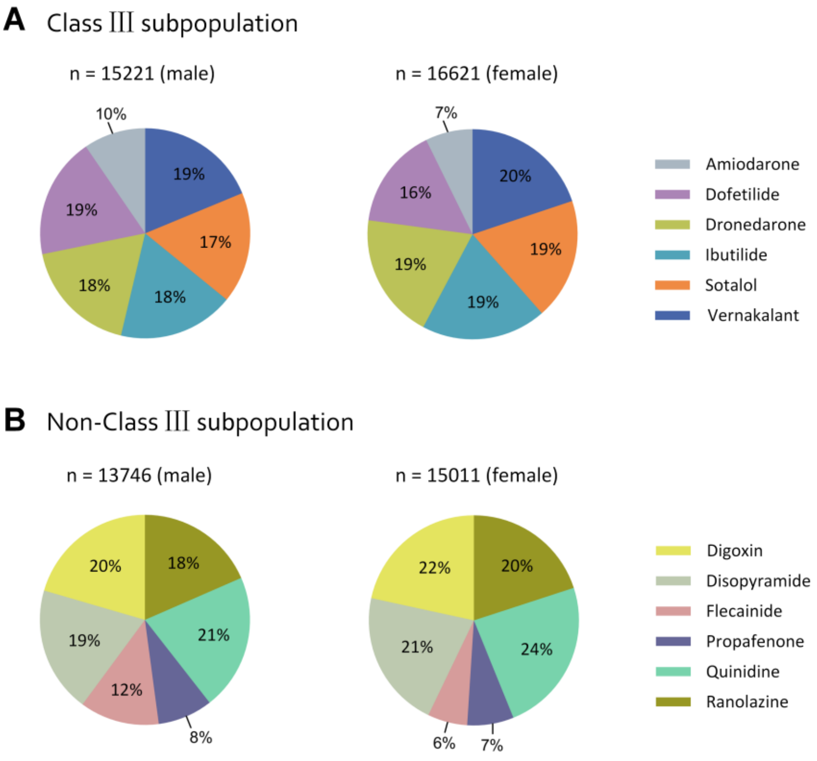

Supplementary Figure S3. *illustrated the distribution of drug populations.* ***(A)*** *detailed the Class III drug population of males, comprising 15,221 action potentials (APs). The distribution of these drugs was as follows: Amiodarone (10%), Dofetilide (19%), Dronedarone (18%), Ibutilide (18%), Sotalol (17%), and Vernakalant (19%). Conversely, the Class III drug population of females included 16,621 APs, with the following proportions: Amiodarone (7%), Dofetilide (16%), Dronedarone (19%), Ibutilide (19%), Sotalol (20%), and Vernakalant (19%).* ***(B)*** *presents the non-Class III drug population of males, totaling 13,746 APs. The composition of this group was: Digoxin (20%), Disopyramide (19%), Flecainide (12%), Propafenone (8%), Quinidine (21%), and Ranolazine (18%). Moreover, the non-Class III drug population for females, consisting of 15,011 APs, is distributed as follows: Digoxin (22%), Disopyramide (21%), Flecainide (6%), Propafenone (7%), Quinidine (24%), and Ranolazine (20%).*

Supplementary Table S4. *The model screening process of constructing drug populations of males and females.*

Drug Class	Drug name	Initial population	Abnormal repolarization	Final population	Drug population
non-Class III	Amiodarone	11,847 (male: 5,663 vs. female: 6,184)	9,162 (male: 4,141 vs female: 5,021)	2,685 (male: 1,522 vs female: 1,163)	31,842 (male:15,221 vs female:16,621)
	Dofetilide	11,847 (male: 5,663 vs. female: 6,184)	5,551 (male: 2,892 vs female: 2,659)	6,296 (male: 2,771 vs female: 3,525)	
	Dronedarone	11,847 (male: 5,663 vs. female: 6,184)	6,098 (male:3,075 vs female:3,023)	5,749 (male:2,588 vs female:3,161)	
	Ibutilide	11,847 (male: 5,663 vs. female: 6,184)	5,950 (male:2,923 vs female:3,027)	5,897 (male:2,740 vs female:3,157)	
	Sotalol	11,847 (male: 5,663 vs. female: 6,184)	6,103 (male:3,076 vs female:3,027)	5,744 (male:2,587 vs female:3,157)	
	Vernakalant	11,847 (male: 5,663 vs.	5,631 (male:2,771 vs	6,216 (male:2,892 vs	

		female: 6,184)	*female:2,860)*	*female:3,324)*	
non-Class III	*Digoxin*	*11,847 (male: 5,663 vs. female: 6,184)*	*5,796 (male:2,914 vs female:2,882)*	*6,051 (male:2,749 vs female:3,302)*	*28,757 (male: 13,746 vs female: 15,011)*
	Disopyramide	*11,847 (male: 5,663 vs. female: 6,184)*	*6,084 (male:3,052 vs female:3,032)*	*5,763 (male:2,611 vs female:3,152)*	
	Flecainide	*11,847 (male: 5,663 vs. female: 6,184)*	*9,159 (male:3,876 vs female:5,283)*	*2,688 (male:1,787 vs female:901)*	
	Propafenone	*11,847 (male: 5,663 vs. female: 6,184)*	*9,559 (male:4,426 vs female:5,133)*	*2,288 (male:1,237 vs female:1,051)*	
	Quinidine	*11,847 (male: 5,663 vs. female: 6,184)*	*5,359 (male:2,777 vs female:2,582)*	*6,488 (male:2,886 vs female:3,602)*	
	Ranolazine	*11,847 (male: 5,663 vs. female: 6,184)*	*6,368 (male:3,187 vs female:3,181)*	*5,479 (male:2,476 vs female:3,003)*	

3. Thirdly, in Figure 9c, there seem to be a lot of features with a very high correlation (absolute value larger than 0.8), especially between ΔRMP and ΔAPA. In this case, the SHAP value wouldn't make a lot of sense among these two data as they are so correlated. The reviewer would suggest the authors do some kind of dimension reduction analysis like PCA to strengthen their claim that "The main difference between Male and Female classifiers was in the ranking of ΔRMP and ΔAPA." Also, the reviewer would expect that the authors wouldn't need all 14 features after PCA to achieve the same/better performance based on how correlated the features are in Figure 9c.

Respond: Thank you for your valuable comment. We understand your concerns regarding the high correlation between certain features and the potential limitations of using SHAP values in such cases.

Regarding the high correlation between ΔRMP and ΔAPA, we agree that this poses a challenge for interpreting the SHAP values. As suggested, we have conducted a dimension reduction analysis such as principal component analysis (PCA). Our PCA analysis showed that indeed a reduced set of features could potentially achieve better performance (See **Table 4**), which is in line with your expectation. This indicates that some of the features may be redundant due to their high correlations.

To further strengthen our claim that "The main difference between Male and Female classifiers was in the ranking of ΔRMP and ΔAPA.", we also conducted ablation experiments. In these experiments, we removed the feature ΔRMP or ΔAPA to illustrate the impact of these features on classification performance (See **Table 5**). The experimental results show that ΔRMP and ΔAPA have different importance in male and female classifiers. In male classifiers, ΔRMP plays a greater role than ΔAPA, while ΔRMP plays a smaller role than ΔAPA in the female classifier. This provides additional evidence to support our claim about the differences in the ranking of these features between male and female classifiers (See **Table 6**).

We appreciate your suggestion and incorporate these analyses into our revised manuscript.

Changes in Text (Pages 10-12, Lines 276- 314)

We also conducted a dimension reduction analysis such as principal component analysis (PCA). The Male model with 12 principal components (AUC: 0.8661 vs. 0.8655) and the Female model with 11 principal components (AUC: 0.8687vs. 0.8663) achieves better performance (See ***Table 4****). These results showed that indeed a reduced set of features could potentially achieve better*

performance.

Table 3. *Performance of SVM using different numbers of top biomarkers based on feature importance ranking shown in Figure 9.*

Type	Number	ACC	SEN	SPE	F1	MCC	AUC
**Sex-special model (Male)**	3	0.7519 (0.7461,0.7577)	0.6406 (0.6306,0.6506)	0.7424 (0.7345,0.7504)	0.6877 (0.6798,0.6957)	0.4876 (0.4753,0.4998)	0.7377 (0.7315,0.7438)
	5	0.8021 (0.7964,0.8079)	0.6781 (0.6677,0.6885)	0.8269 (0.8177,0.8362)	0.7451 (0.7371,0.7530)	0.5938 (0.5817,0.6060)	0.7862 (0.7802,0.7923)
	7	0.8590 (0.8543,0.8637)	0.7285 (0.7179,0.7391)	0.9250 (0.9192,0.9309)	0.8150 (0.8079,0.8221)	0.7169 (0.7075,0.7263)	0.8423 (0.8369,0.8476)
	9	0.8694 (0.8612,0.8776)	0.7446 (0.7339,0.7552)	0.9364 (0.9198,0.9530)	0.8294 (0.8191,0.8398)	0.7385 (0.7206,0.7565)	0.8534 (0.8452,0.8616)
	11	0.8738 (0.8674,0.8802)	0.7530 (0.7414,0.7646)	0.9391 (0.9265,0.9517)	0.8357 (0.8272,0.8443)	0.7472 (0.7336,0.7608)	0.8583 (0.8516,0.8650)
	**14**	**0.8802 (0.8751,0.8853)**	**0.7635 (0.7569,0.7701)**	**0.9454 (0.9320,0.9587)**	**0.8450 (0.8389,0.8510)**	**0.7620 (0.7478,0.7762)**	**0.8655 (0.8607,0.8703)**
**Sex-special model (Female)**	3	0.7299 (0.7254,0.7344)	0.6576 (0.6513,0.6638)	0.7129 (0.7066,0.7192)	0.6841 (0.6789,0.6893)	0.4501 (0.4409,0.4592)	0.7227 (0.7182,0.7272)
	5	0.7843 (0.7783,0.7902)	0.7116 (0.7027,0.7204)	0.7834 (0.7771,0.7898)	0.7458 (0.7382,0.7533)	0.5612 (0.5490,0.5735)	0.7770 (0.7708,0.7833)
	7	0.8511 (0.8464,0.8558)	0.7395 (0.7301,0.7489)	0.9088 (0.9020,0.9155)	0.8154 (0.8090,0.8219)	0.7033 (0.6938,0.7128)	0.8400 (0.8350,0.8450)
	9	0.8635 (0.8578,0.8692)	0.7460 (0.7388,0.7532)	0.9338 (0.9231,0.9446)	0.8294 (0.8224,0.8363)	0.7306 (0.7184,0.7429)	0.8518 (0.8461,0.8575)
	11	0.8703 (0.8656,0.8750)	0.7569 (0.7492,0.7646)	0.9398 (0.9332,0.9463)	0.8385 (0.8323,0.8446)	0.7443 (0.7346,0.7539)	0.8590 (0.8541,0.8639)
	**14**	**0.8774 (0.8738,0.8809)**	**0.7660 (0.7613,0.7707)**	**0.9484 (0.9442,0.9525)**	**0.8475 (0.8429,0.8520)**	**0.7601 (0.7509,0.7691)**	**0.8663 (0.8626,0.8699)**
**Non-sex-special model**	3	0.7337 (0.7269,0.7404)	0.8072 (0.8020,0.8124)	0.7262 (0.7196,0.7328)	0.7646 (0.7590,0.7701)	0.4634 (0.4497,0.4771)	0.7280 (0.7210,0.7349)
	5	0.7512 (0.7453,0.7571)	0.8115 (0.8034,0.8195)	0.7463 (0.7417,0.7508)	0.7775 (0.7718,0.7832)	0.4988 (0.4867,0.5109)	0.7466 (0.7407,0.7524)
	7	0.7891 (0.7853,0.7930)	0.8097 (0.8033,0.8161)	0.7993 (0.7946,0.8041)	0.8045 (0.8007,0.8082)	0.5759 (0.5680,0.5837)	0.7876 (0.7837,0.7915)
	9	0.8029 (0.7993,0.8066)	0.8161 (0.8108,0.8215)	0.8161 (0.8100,0.8222)	0.8161 (0.8130,0.8192)	0.6040 (0.5964,0.6115)	0.8019 (0.7980,0.8058)
	11	0.8041 (0.8025,0.8058)	0.8163 (0.8078,0.8248)	0.8178 (0.8140,0.8217)	0.8170 (0.8144,0.8197)	0.6065 (0.6034,0.6095)	0.8032 (0.8019,0.8045)
	**14**	**0.8095 (0.8064,0.8127)**	**0.8253 (0.8187,0.8319)**	**0.8217 (0.8177,0.8257)**	**0.8226 (0.8193,0.8259)**	**0.6179 (0.6101,0.6258)**	**0.8085 (0.8053,0.8117)**

*We also conducted a dimension reduction analysis such as principal component analysis (PCA). The Male model with 12 principal components (AUC: 0.8661 vs. 0.8655) and the Female model with 11 principal components (AUC: 0.8687vs. 0.8663) achieves better performance (See **Table 4**). These results showed that indeed a reduced set of features could potentially achieve better performance.*

Table 4. *Principal Components (PCs) Analysis Coupled with Support Vector Machine (SVM) Classification Utilizing Sex-Specific Classifiers (Male and Female) for Discriminating Class III Drugs from Non-Class III Drugs.*

Type	Number	ACC	SEN	SPE	F1	AUC	MCC
**Sex-special model (Male) (PCA)**	3	0.7594 (0.7561,0.7627)	0.6834 (0.6729,0.6939)	0.7329 (0.7278,0.7381)	0.7072 (0.7018,0.7126)	0.7495 (0.7457,0.7533)	0.5044 (0.4974,0.5114)
	5	0.8005 (0.7975,0.8035)	0.6956 (0.6833,0.7079)	0.8088 (0.8035,0.8141)	0.7478 (0.7419,0.7537)	0.7869 (0.7828,0.7909)	0.5890 (0.5828,0.5953)
	7	0.8120 (0.8080,0.8161)	0.7151 (0.7004,0.7298)	0.8201 (0.8135,0.8267)	0.7639 (0.7568,0.7710)	0.7994 (0.7943,0.8046)	0.6131 (0.6048,0.6214)
	9	0.8376 (0.8347,0.8405)	0.7221 (0.7121,0.7321)	0.8744 (0.8692,0.8797)	0.7909 (0.7859,0.7959)	0.8226 (0.8189,0.8263)	0.6684 (0.6626,0.6742)
	10	0.8725 (0.8691,0.8759)	0.7470 (0.7371,0.7569)	0.9412 (0.9377,0.9446)	0.8328 (0.8274,0.8383)	0.8562 (0.8520,0.8604)	0.7448 (0.7385,0.7512)
	11	0.8815 (0.8760,0.8871)	0.76243 (0.7506,0.7741)	0.9491 (0.9432,0.9550)	0.8455 (0.8375,0.8535)	0.8660 (0.8598,0.8723)	0.7632 (0.7522,0.7743)
	**12**	**0.8816 (0.8761,0.8870)**	**0.7625 (0.7509,0.7741)**	**0.9491 (0.9432,0.9550)**	**0.8456 (0.8377,0.8535)**	**0.8661 (0.8599,0.8723)**	**0.7633 (0.7524,0.7743)**
	13	0.8816 (0.8761,0.8870)	0.7625 (0.7509,0.7741)	0.9491 (0.9432,0.9550)	0.8456 (0.8377,0.8535)	0.8661 (0.8599,0.8723)	0.7633 (0.7524,0.7743)
	14	0.8816 (0.8761,0.8870)	0.7625 (0.7509,0.7741)	0.9491 (0.9432,0.9550)	0.8456 (0.8377,0.8535)	0.8661 (0.8599,0.8723)	0.7633 (0.7524,0.7743)
**Sex-special**	3	0.7520 (0.7417,0.7624)	0.6739 (0.6578,0.6899)	0.7467 (0.7349,0.7585)	0.7084 (0.6950,0.7217)	0.7445 (0.7337,0.7554)	0.4958 (0.4744,0.5171)
	5	0.7944	0.7051	0.8105	0.7541	0.7858	0.5831

model (Female) (PCA)		(0.7858,0.8030)	(0.6944,0.7158)	(0.7969,0.8241)	(0.7440,0.7641)	(0.7772,0.7944)	(0.5652,0.6010)
	7	0.8048 (0.7971,0.8126)	0.7137 (0.7061,0.7213)	0.8263 (0.8107,0.8420)	0.7658 (0.7577,0.7739)	0.7961 (0.7887,0.8035)	0.6049 (0.5884,0.6215)
	9	0.8493 (0.8420,0.8566)	0.7327 (0.7259,0.7395)	0.9130 (0.9010,0.9250)	0.8130 (0.8042,0.8217)	0.8381 (0.8308,0.8453)	0.7010 (0.6854,0.7166)
	10	0.8529 (0.8437,0.8622)	0.7352 (0.7251,0.7452)	0.9198 (0.9060,0.9337)	0.8172 (0.8058,0.8286)	0.8417 (0.8323,0.8510)	0.7090 (0.6894,0.7287)
	**11**	**0.8793 (0.8744,0.8842)**	**0.7680 (0.7604,0.7756)**	**0.9529 (0.9480,0.9577)**	**0.8505 (0.8441,0.8569)**	**0.8687 (0.8635,0.8738)**	**0.7636 (0.7537,0.7735)**
	12	0.8793 (0.8744,0.8842)	0.7680 (0.7604,0.7756)	0.9529 (0.9480,0.9577)	0.8505 (0.8441,0.8569)	0.8687 (0.8635,0.8738)	0.7636 (0.7537,0.7735)
	13	0.8793 (0.8744,0.8842)	0.7680 (0.7604,0.7756)	0.9529 (0.9480,0.9577)	0.8505 (0.8441,0.8569)	0.8687 (0.8635,0.8738)	0.7636 (0.7537,0.7735)
	14	0.8793 (0.8744,0.8842)	0.7680 (0.7604,0.7756)	0.9529 (0.9480,0.9577)	0.8505 (0.8441,0.8569)	0.8687 (0.8635,0.8738)	0.7636 (0.7537,0.7735)

*We also conducted ablation experiments. In these experiments, we removed the feature ΔRMP or ΔAPA to illustrate the impact of these features on classification performance (See **Table 5**). The experimental results show that ΔRMP and ΔAPA have different importance in male and female classifiers. In male classifiers, ΔRMP plays a greater role than ΔAPA, while ΔRMP plays a smaller role than ΔAPA in the female classifier. This provides additional evidence to support our claim about the differences in the ranking of these features between male and female classifiers (See **Table 6**).*

Table 5. *Feature ablation experimental results.*

Type	features	ACC	SEN	SPE	F1	AUC	MCC
**Sex-special model (Male)**	W/oΔRMP	0.8437 (0.8347,0.8526)	0.7316 (0.7206,0.7427)	0.8823 (0.8609,0.9036)	0.7997 (0.7897,0.8097)	0.8293 (0.8210,0.8376)	0.6815 (0.6620,0.7010)
	W/oΔAPA	0.8542 (0.8477,0.8606)	0.7421 (0.7384,0.7458)	0.8986 (0.8831,0.9142)	0.8128 (0.8059,0.8198)	0.8398 (0.8340,0.8456)	0.7038 (0.6895,0.7182)
	-	0.8802 (0.8751,0.8853)	0.7635 (0.7569,0.7701)	0.9454 (0.9320,0.9587)	0.8450 (0.8389,0.8510)	0.8659 (0.8607,0.8703)	0.7620 (0.7478,0.7762)
**Sex-special model (Female)**	W/oΔRMP	0.8374 (0.8313,0.8434)	0.7485 (0.7379,0.7591)	0.8677 (0.8582,0.8772)	0.8037 (0.7960,0.8113)	0.8285 (0.8223,0.8348)	0.6715 (0.6591,0.6840)
	W/oΔAPA	0.8234 (0.8193,0.8274)	0.7210 (0.7159,0.7262)	0.8594 (0.8490,0.8697)	0.7841 (0.7801,0.7881)	0.8132 (0.8095,0.8169)	0.6437 (0.6348,0.6526)
	-	0.8774 (0.8738,0.8809)	0.7660 (0.7613,0.7707)	0.9484 (0.9442,0.9525)	0.8475 (0.8429,0.8520)	0.8663 (0.8626,0.8699)	0.7601 (0.7509,0.7691)

Table 6. *Role of ΔRMP or ΔAPA in the sex-special models.*

Type	features	ΔACC (%)	ΔSEN (%)	ΔSPE (%)	ΔF1 (%)	ΔAUC (%)	ΔMCC (%)
**Sex-special model (Male)**	**W/oΔRMP**	**3.7%**	**3.2%**	**6.3%**	**4.5%**	**3.7%**	**8.1%**
	W/oΔAPA	2.6%	2.1%	4.7%	3.2%	2.6%	5.8%
**Sex-special model (Female)**	W/oΔRMP	4.0%	1.8%	8.1%	4.4%	3.8%	8.9%
	**W/oΔAPA**	**5.4%**	**4.5%**	**8.9%**	**6.3%**	**5.3%**	**11.6%**

4. Minor Points: The citation style in Page 5 line 23 is inconsistent with the rest of the paper.

Respond: Thank you for pointing out this inconsistency. We apologize for the oversight. We have carefully reviewed and ensured that the citation style throughout the paper is consistent in the revised manuscript.

Changes in Text (Pages 25-30, Lines 748- 977)

1 *Elliott, A. D., Middeldorp, M. E., Van Gelder, I. C., Albert, C. M. & Sanders, P. Epidemiology and modifiable risk factors for atrial fibrillation. Nature reviews. Cardiology* ***20****, 404-417, doi:10.1038/s41569-022-00820-8 (2023).*

2 *Weberndörfer, V. et al. Sex differences in rate and rhythm control for atrial fibrillation. Europace : European pacing, arrhythmias, and cardiac electrophysiology : journal of the working groups on cardiac pacing, arrhythmias, and cardiac cellular electrophysiology of the European Society of Cardiology* ***21****, 690-697, doi:10.1093/europace/euy295 (2019).*

3 *Vogel, B. et al. The Lancet women and cardiovascular disease Commission: reducing the global burden by 2030. Lancet (London, England)* ***397****, 2385-2438, doi:10.1016/s0140-6736(21)00684-x (2021).*

4 Regitz-Zagrosek, V. & Gebhard, C. Gender medicine: effects of sex and gender on cardiovascular disease manifestation and outcomes. Nature reviews. Cardiology **20**, 236-247, doi:10.1038/s41569-022-00797-4 (2023).

5 Wyse, D. G. et al. A comparison of rate control and rhythm control in patients with atrial fibrillation. The New England journal of medicine **347**, 1825-1833, doi:10.1056/NEJMoa021328 (2002).

6 Kirchhof, P. et al. Early Rhythm-Control Therapy in Patients with Atrial Fibrillation. The New England journal of medicine **383**, 1305-1316, doi:10.1056/NEJMoa2019422 (2020).

7 Van Gelder, I. C. et al. A comparison of rate control and rhythm control in patients with recurrent persistent atrial fibrillation. The New England journal of medicine **347**, 1834-1840, doi:10.1056/NEJMoa021375 (2002).

8 Heijman, J., Hohnloser, S. H. & Camm, A. J. Antiarrhythmic drugs for atrial fibrillation: lessons from the past and opportunities for the future. Europace : European pacing, arrhythmias, and cardiac electrophysiology : journal of the working groups on cardiac pacing, arrhythmias, and cardiac cellular electrophysiology of the European Society of Cardiology **23**, ii14-ii22, doi:10.1093/europace/euaa426 (2021).

9 Saljic, A., Heijman, J. & Dobrev, D. Recent Advances in Antiarrhythmic Drug Therapy. Drugs **83**, 1147-1160, doi:10.1007/s40265-023-01923-3 (2023).

10 Dobrev, D., Aguilar, M., Heijman, J., Guichard, J. B. & Nattel, S. Postoperative atrial fibrillation: mechanisms, manifestations and management. Nature reviews. Cardiology **16**, 417-436, doi:10.1038/s41569-019-0166-5 (2019).

11 Dan, G. A. et al. Antiarrhythmic drugs-clinical use and clinical decision making: a consensus document from the European Heart Rhythm Association (EHRA) and European Society of Cardiology (ESC) Working Group on Cardiovascular Pharmacology, endorsed by the Heart Rhythm Society (HRS), Asia-Pacific Heart Rhythm Society (APHRS) and International Society of Cardiovascular Pharmacotherapy (ISCP). Europace : European pacing, arrhythmias, and cardiac electrophysiology : journal of the working groups on cardiac pacing, arrhythmias, and cardiac cellular electrophysiology of the European Society of Cardiology **20**, 731-732an, doi:10.1093/europace/eux373 (2018).

12 Preliminary report: effect of encainide and flecainide on mortality in a randomized trial of arrhythmia suppression after myocardial infarction. The New England journal of medicine **321**, 406-412, doi:10.1056/nejm198908103210629 (1989).

13 Wybraniec, M. T. et al. Antiarrhythmic drugs for pharmacological cardioversion of atrial fibrillation and sex differences: Insights from the CANT II Study. Kardiologia polska **81**, 1089-1095, doi:10.33963/v.kp.97392 (2023).

14 Tsikouris, J. P. & Cox, C. D. A review of class III antiarrhythmic agents for atrial fibrillation: maintenance of normal sinus rhythm. Pharmacotherapy **21**, 1514-1529, doi:10.1592/phco.21.20.1514.34484 (2001).

15 Lei, M., Wu, L., Terrar, D. A. & Huang, C. L. Modernized Classification of Cardiac Antiarrhythmic Drugs. Circulation **138**, 1879-1896, doi:10.1161/circulationaha.118.035455 (2018).

16 Dixit, S., Oranefo, J. U. & Raad, M. Atrial Fibrillation Management With Antiarrhythmic Drug Therapy: Balancing Benefits and Bradycardia Risks. Journal of the American College of Cardiology **83**, 1039-1041, doi:10.1016/j.jacc.2024.01.015 (2024).

17 Font, C. M. & Tran, C. T. Class III Antiarrhythmics and Periprocedural Torsades de Pointes. JACC. Case reports **23**, 101998, doi:10.1016/j.jaccas.2023.101998 (2023).

18 Britton, O. J. et al. Experimentally calibrated population of models predicts and explains intersubject variability in cardiac cellular electrophysiology. Proceedings of the National Academy of Sciences of the United States of America **110**, E2098-2105, doi:10.1073/pnas.1304382110 (2013).

19 Kernik, D. C. et al. A computational model of induced pluripotent stem-cell derived cardiomyocytes incorporating experimental variability from multiple data sources. The Journal of physiology **597**, 4533-4564, doi:10.1113/jp277724 (2019).

20 Muszkiewicz, A. et al. Variability in cardiac electrophysiology: Using experimentally-calibrated populations of models to move beyond the single virtual physiological human paradigm. Progress in biophysics and molecular biology **120**, 115-127, doi:10.1016/j.pbiomolbio.2015.12.002 (2016).

21 Niederer, S. A. et al. Creation and application of virtual patient cohorts of heart models. Philosophical transactions. Series A,

Mathematical, physical, and engineering sciences **378**, 20190558, doi:10.1098/rsta.2019.0558 (2020).

22 Sánchez, C. et al. Inter-subject variability in human atrial action potential in sinus rhythm versus chronic atrial fibrillation. *PloS one* **9**, e105897, doi:10.1371/journal.pone.0105897 (2014).

23 Lawson, B. A. J. et al. Unlocking data sets by calibrating populations of models to data density: A study in atrial electrophysiology. *Science advances* **4**, e1701676, doi:10.1126/sciadv.1701676 (2018).

24 Passini, E. et al. Human In Silico Drug Trials Demonstrate Higher Accuracy than Animal Models in Predicting Clinical Pro-Arrhythmic Cardiotoxicity. *Frontiers in physiology* **8**, 668, doi:10.3389/fphys.2017.00668 (2017).

25 Lancaster, M. C. & Sobie, E. A. Improved Prediction of Drug-Induced Torsades de Pointes Through Simulations of Dynamics and Machine Learning Algorithms. *Clinical pharmacology and therapeutics* **100**, 371-379, doi:10.1002/cpt.367 (2016).

26 Chang, K. C. et al. Uncertainty Quantification Reveals the Importance of Data Variability and Experimental Design Considerations for in Silico Proarrhythmia Risk Assessment. *Frontiers in physiology* **8**, 917, doi:10.3389/fphys.2017.00917 (2017).

27 Passini, E. et al. Drug-induced shortening of the electromechanical window is an effective biomarker for in silico prediction of clinical risk of arrhythmias. *British journal of pharmacology* **176**, 3819-3833, doi:10.1111/bph.14786 (2019).

28 Li, Z. et al. General Principles for the Validation of Proarrhythmia Risk Prediction Models: An Extension of the CiPA In Silico Strategy. *Clinical pharmacology and therapeutics* **107**, 102-111, doi:10.1002/cpt.1647 (2020).

29 Ni, H. et al. Populations of in silico myocytes and tissues reveal synergy of multiatrial-predominant K(+) -current block in atrial fibrillation. *British journal of pharmacology* **177**, 4497-4515, doi:10.1111/bph.15198 (2020).

30 Llopis-Lorente, J., Trenor, B. & Saiz, J. Considering population variability of electrophysiological models improves the in silico assessment of drug-induced torsadogenic risk. *Computer methods and programs in biomedicine* **221**, 106934, doi:10.1016/j.cmpb.2022.106934 (2022).

31 Hwang, M., Lim, C. H., Leem, C. H. & Shim, E. B. In silico models for evaluating proarrhythmic risk of drugs. *APL bioengineering* **4**, 021502, doi:10.1063/1.5132618 (2020).

32 Adedinsewo, D. A. et al. Cardiovascular Disease Screening in Women: Leveraging Artificial Intelligence and Digital Tools. *Circulation research* **130**, 673-690, doi:10.1161/circresaha.121.319876 (2022).

33 Romero, P. et al. Clinically-Driven Virtual Patient Cohorts Generation: An Application to Aorta. *Frontiers in physiology* **12**, 713118, doi:10.3389/fphys.2021.713118 (2021).

34 Llopis-Lorente, J. et al. In Silico Classifiers for the Assessment of Drug Proarrhythmicity. *Journal of chemical information and modeling* **60**, 5172-5187, doi:10.1021/acs.jcim.0c00201 (2020).

35 Dasí, A. et al. What determines the optimal pharmacological treatment of atrial fibrillation? Insights from in silico trials in 800 virtual atria. *The Journal of physiology* **601**, 4013-4032, doi:10.1113/jp284730 (2023).

36 Hernandez-Hernandez, G. et al. A computational model predicts sex-specific responses to calcium channel blockers in mammalian mesenteric vascular smooth muscle. *eLife* **12**, doi:10.7554/eLife.90604 (2024).

37 Llopis-Lorente, J. et al. Combining pharmacokinetic and electrophysiological models for early prediction of drug-induced arrhythmogenicity. *Computer methods and programs in biomedicine* **242**, 107860, doi:10.1016/j.cmpb.2023.107860 (2023).

38 Peirlinck, M., Sahli Costabal, F. & Kuhl, E. Sex Differences in Drug-Induced Arrhythmogenesis. *Frontiers in physiology* **12**, 708435, doi:10.3389/fphys.2021.708435 (2021).

39 Zhang, X. et al. Enhanced Ca2+-Driven Arrhythmias in Female Patients with Atrial Fibrillation: Insights from Computational Modeling. *bioRxiv : the preprint server for biology*, doi:10.1101/2024.03.04.583217 (2024).

40 Yang, P. C. & Clancy, C. E. In silico Prediction of Sex-Based Differences in Human Susceptibility to Cardiac Ventricular Tachyarrhythmias. *Frontiers in physiology* **3**, 360, doi:10.3389/fphys.2012.00360 (2012).

41 Fogli Iseppe, A. et al. Sex-Specific Classification of Drug-Induced Torsade de Pointes Susceptibility Using Cardiac Simulations and Machine Learning. *Clinical pharmacology and therapeutics* **110**, 380-391, doi:10.1002/cpt.2240 (2021).

42 Morotti, S. et al. Quantitative cross-species translators of cardiac myocyte electrophysiology: Model training, experimental

validation, and applications. Science advances **7**, eabg0927, doi:10.1126/sciadv.abg0927 (2021).

43 Kurokawa, J., Kodama, M., Clancy, C. E. & Furukawa, T. Sex hormonal regulation of cardiac ion channels in drug-induced QT syndromes. Pharmacology & therapeutics **168**, 23-28, doi:10.1016/j.pharmthera.2016.09.004 (2016).

44 Matsumoto, C. et al. The formation of K(V)2.1 macro-clusters is required for sex-specific differences in L-type Ca(V)1.2 clustering and function in arterial myocytes. Communications biology **6**, 1165, doi:10.1038/s42003-023-05527-1 (2023).

45 Yang, P. C. et al. A multiscale computational modelling approach predicts mechanisms of female sex risk in the setting of arousal-induced arrhythmias. The Journal of physiology **595**, 4695-4723, doi:10.1113/jp273142 (2017).

46 Hellgren, K. T., Ni, H., Morotti, S. & Grandi, E. Predictive Male-to-Female Translation of Cardiac Electrophysiological Response to Drugs. JACC. Clinical electrophysiology **9**, 2642-2648, doi:10.1016/j.jacep.2023.08.016 (2023).

47 Pecha, S. et al. Resting membrane potential is less negative in trabeculae from right atrial appendages of women, but action potential duration does not shorten with age. Journal of molecular and cellular cardiology **176**, 1-10, doi:10.1016/j.yjmcc.2023.01.006 (2023).

48 Sims, C. et al. Sex, age, and regional differences in L-type calcium current are important determinants of arrhythmia phenotype in rabbit hearts with drug-induced long QT type 2. Circulation research **102**, e86-100, doi:10.1161/circresaha.108.173740 (2008).

49 James, A. F., Arberry, L. A. & Hancox, J. C. Gender-related differences in ventricular myocyte repolarization in the guinea pig. Basic research in cardiology **99**, 183-192, doi:10.1007/s00395-003-0451-6 (2004).

50 Wu, Y. & Anderson, M. E. Reduced repolarization reserve in ventricular myocytes from female mice. Cardiovascular research **53**, 763-769, doi:10.1016/s0008-6363(01)00387-x (2002).

51 Barajas-Martinez, H. et al. Larger dispersion of INa in female dog ventricle as a mechanism for gender-specific incidence of cardiac arrhythmias. Cardiovascular research **81**, 82-89, doi:10.1093/cvr/cvn255 (2009).

52 Trépanier-Boulay, V., St-Michel, C., Tremblay, A. & Fiset, C. Gender-based differences in cardiac repolarization in mouse ventricle. Circulation research **89**, 437-444, doi:10.1161/hh1701.095644 (2001).

53 Chen, G., Yang, X., Alber, S., Shusterman, V. & Salama, G. Regional genomic regulation of cardiac sodium-calcium exchanger by oestrogen. The Journal of physiology **589**, 1061-1080, doi:10.1113/jphysiol.2010.203398 (2011).

54 Yan, S. et al. Bisphenol A and 17β-estradiol promote arrhythmia in the female heart via alteration of calcium handling. PloS one **6**, e25455, doi:10.1371/journal.pone.0025455 (2011).

55 Laasmaa, M. et al. Cardiomyocytes from female compared to male mice have larger ryanodine receptor clusters and higher calcium spark frequency. The Journal of physiology **601**, 4033-4052, doi:10.1113/jp284515 (2023).

56 Herraiz-Martínez, A. et al. Influence of sex on intracellular calcium homoeostasis in patients with atrial fibrillation. Cardiovascular research **118**, 1033-1045, doi:10.1093/cvr/cvab127 (2022).

57 Ahmad, G. N., Fatima, H., Ullah, S., Saidi, A. S. & Imdadullah. Efficient Medical Diagnosis of Human Heart Diseases Using Machine Learning Techniques With and Without GridSearchCV. IEEE Access **10**, 80151-80173, doi:10.1109/ACCESS.2022.3165792 (2022).

58 Xiao, L., Zhang, L., Han, W., Wang, Z. & Nattel, S. Sex-based transmural differences in cardiac repolarization and ionic-current properties in canine left ventricles. American journal of physiology. Heart and circulatory physiology **291**, H570-580, doi:10.1152/ajpheart.01288.2005 (2006).

59 Liu, X. K. et al. Gender difference in the cycle length-dependent QT and potassium currents in rabbits. The Journal of pharmacology and experimental therapeutics **285**, 672-679 (1998).

60 Mason, S. A. & MacLeod, K. T. Cardiac action potential duration and calcium regulation in males and females. Biochemical and Biophysical Research Communications **388**, 565-570, doi:https://doi.org/10.1016/j.bbrc.2009.08.050 (2009).

61 Tanaka, M. et al. Sex differences and arrhythmia recurrence after catheter ablation for atrial fibrillation. European Heart Journal **41**, doi:10.1093/ehjci/ehaa946.0601 (2020).

62 Park, Y. J. et al. Sex difference in atrial fibrillation recurrence after catheter ablation and antiarrhythmic drugs. Heart

(British Cardiac Society) **109**, 519-526, doi:10.1136/heartjnl-2021-320601 (2023).

63 Matthews, E. J. & Frid, A. A. Prediction of drug-related cardiac adverse effects in humans--A: creation of a database of effects and identification of factors affecting their occurrence. Regulatory toxicology and pharmacology : RTP **56**, 247-275, doi:10.1016/j.yrtph.2009.11.006 (2010).

64 Bai, J. et al. In Silico Assessment of Class I Antiarrhythmic Drug Effects on Pitx2-Induced Atrial Fibrillation: Insights from Populations of Electrophysiological Models of Human Atrial Cells and Tissues. International journal of molecular sciences **22**, doi:10.3390/ijms22031265 (2021).

65 Shan, Y., Cheung, L., Zhou, Y., Huang, Y. & Huang, R. S. A systematic review on sex differences in adverse drug reactions related to psychotropic, cardiovascular, and analgesic medications. Frontiers in pharmacology **14**, 1096366, doi:10.3389/fphar.2023.1096366 (2023).

66 Dasí, A. et al. In Silico TRials guide optimal stratification of ATrIal FIbrillation patients to Catheter Ablation and pharmacological medicaTION: the i-STRATIFICATION study. Europace : European pacing, arrhythmias, and cardiac electrophysiology : journal of the working groups on cardiac pacing, arrhythmias, and cardiac cellular electrophysiology of the European Society of Cardiology **26**, doi:10.1093/europace/euae150 (2024).

67 Jiao, L. et al. Estrogen and calcium handling proteins: new discoveries and mechanisms in cardiovascular diseases. American journal of physiology. Heart and circulatory physiology **318**, H820-h829, doi:10.1152/ajpheart.00734.2019 (2020).

68 Gori, M. et al. Sex-specific cardiovascular structure and function in heart failure with preserved ejection fraction. European journal of heart failure **16**, 535-542, doi:10.1002/ejhf.67 (2014).

69 Grandi, E. et al. Human atrial action potential and Ca2+ model: sinus rhythm and chronic atrial fibrillation. Circulation research **109**, 1055-1066, doi:10.1161/circresaha.111.253955 (2011).

70 Dasí, A. et al. In-silico drug trials for precision medicine in atrial fibrillation: From ionic mechanisms to electrocardiogram-based predictions in structurally-healthy human atria. Frontiers in physiology **13**, 966046, doi:10.3389/fphys.2022.966046 (2022).

71 D.K, T., B.G, P. & Xiong, F. Auto-detection of epileptic seizure events using deep neural network with different feature scaling techniques. Pattern Recognition Letters **128**, 544-550, doi:https://doi.org/10.1016/j.patrec.2019.10.029 (2019).

72 Sah, S., Surendiran, B., Dhanalakshmi, R. & Yamin, M. Covid-19 cases prediction using SARIMAX Model by tuning hyperparameter through grid search cross-validation approach. Expert Systems **40**, e13086, doi:https://doi.org/10.1111/exsy.13086 (2023).

73 Luque, A., Carrasco, A., Martín, A. & de las Heras, A. The impact of class imbalance in classification performance metrics based on the binary confusion matrix. Pattern Recognition **91**, 216-231, doi:https://doi.org/10.1016/j.patcog.2019.02.023 (2019).

Reviewer #3:

1. The manuscript claims to introduce a novel pipeline, but the methodology heavily relies on a previously established multivariable analysis technique (cited in [47]), and is based on the hypothesis that sex is a critical factor in drug response, yet it fails to identify any new biomarkers beyond the 14 previously known.

Respond: We appreciate your comment regarding the methodology of our study. Acknowledging that our method is an existing method, we emphasize that the focus of this paper is on the impact of gender factors on drug efficacy and the underlying mechanisms. Our aim was not necessarily to introduce a completely novel methodology in terms of the analysis technique itself. Instead, we utilized this existing approach to explore a new aspect of drug response - the role of gender. By integrating in vitro measurements, in silico models, and machine learning tools in a unique way, we were able to specifically address the problem of gender-specific drug response in atrial fibrillation. This combination of approaches provides a more comprehensive understanding of the complex interactions between drugs, gender, and cardiac electrophysiology.

It should be noted here that there are only 7 biomarkers in vitro measurements. We obtained another 7 biomarkers through in silico model simulation, thus realizing the classification task with machine learning models based on 14 biomarkers. This process was crucial in enabling a more comprehensive analysis. The in vitro measurements provided direct empirical data, while the computer model simulation allowed us to expand the range of variables considered and gain insights into aspects that might not be easily measured directly. Together, these 14 biomarkers formed the basis for our more accurate assessment of drug responses in the context of gender differences.

Regarding the biomarkers, we understand the concern about not identifying new ones beyond the 14 previously known. Our study's objective was not centered on biomarker discovery per se. Instead, we aimed to highlight the significance of gender differences in drug classification using these established biomarkers. The 14 biomarkers have been well-documented in previous research, and our contribution lies in demonstrating their relevance and importance in a gender-specific context. Our analysis provides insights into how these biomarkers can be utilized to develop more accurate classifiers for antiarrhythmic drugs.

In the revised manuscript, we have not only summarized the main contributions of this study but also engaged in a more in-depth discussion about the potential limitations related to biomarkers.

Changes in Text ***(Page 13, Lines 343- 356, Page 17, Lines 491- 502)***

Our main findings included: (1) Significant differences in AP and CT biomarkers between virtual male and female patients within the drug population; (2) AP biomarkers were more important than CT biomarkers in drug classification, though CT biomarkers were essential for optimal classification accuracy; (3) ΔAPA and ΔRMP were identified as key features reflecting differences between male- and female-specific classifiers; (4) Sex-specific classifiers outperformed non-sex-specific classifiers, while sex-biased classifiers were inferior to sex-specific classifiers; and (5) The sex-dependent differences in drug response may be partly explained by lower levels of IK1, INa, and Ito in female versus male patients. These results underscore the importance of incorporating sex differences in the evaluation of AAD efficacy and emphasize the potential for personalized medicine approaches in treating AF based on AAD classification. This study

indicates the value of combining mechanistic simulations with ML and provides insight into the role of gender factor in the drug efficacy evaluation.

Thirdly, the cellular electrophysiological mechanisms of AF mainly include calcium disorder and action potential shortening. However, ex vivo experimental data used to calibrate the AF population do not include data related to calcium disorder. Therefore, we did not use a complex model of human atrial cardiomyocyte electrophysiology and calcium handling with biochemically detailed systems. It is important to emphasize that the gender differences in calcium disorder should also be considered in future research. Finally, the electrophysiological phenomena of single atrial cells can only partially reflect the reentrant arrhythmias at the organ level in AF. At the organ level, AF may involve gender differences in cardiac anatomical structure, the degree and distribution of fibrosis, and potential organ-level electrical heterogeneity[68]. Therefore, comprehensive gender differences in the evaluation of drug efficacy and safety require more in-depth research.

2. - The classification task is restricted to a binary outcome (Class III vs. non-Class III drugs), which is overly simplistic for drug discovery applications. More nuanced classifications, such as drug efficacy and safety across multiple dimensions, would be more impactful and relevant.

Respond: We appreciate your comment. Since drugs used in atrial fibrillation are mainly Class III and Class I (non-Class III) drugs. Therefore, this led to our binary classification task. The binary classification was a starting point to show gender differences in drug response for atrial fibrillation drugs (mainly Class III and Class I). We agree more nuanced classifications are better for drug discovery. In our previous research (*"Bai J, Zhu Y, Lo A, et al. In Silico Assessment of Class I Antiarrhythmic Drug Effects on Pitx2-Induced Atrial Fibrillation: Insights from Populations of Electrophysiological Models of Human Atrial Cells and Tissues. Int J Mol Sci. 2021;22(3):1265. Published 2021 Jan 27. doi:10.3390/ijms22031265 Zhu Y, Bai J, Lo A, Lu Y, Zhao J. Mechanisms underlying pro-arrhythmic abnormalities arising from Pitx2-induced electrical remodelling: an in silico intersubject variability study. Ann Transl Med. 2021;9(2):106. doi:10.21037/atm-20-5660"*), we've studied drug safety before. Our future focus is on exploring drug efficacy and safety across multiple dimensions. We'll look for additional biomarkers and features related to arrhythmia suppression, adverse events, and long-term patient outcomes. In the revised manuscript, we have discussed this thoroughly and suggest ways to expand our classification framework.

Changes in Text (Pages 16-17, Lines 464-502)

Firstly, the drugs used in this study are divided into two categories (Class III and non-Class III), though these drugs fall into more than four categories according to the Vaughan Williams classification system. This focus is due to the widespread use of Class III drugs in AF and their potential risk of inducing TdP, a specific form of polymorphic ventricular tachycardia that can precipitate ventricular fibrillation and cause sudden cardiac death. From the perspective of clinical applications, further research needs to investigate more detailed classification methods. In future work, we plan to expand our drug classification scope to include more drug categories and explore more refined classification criteria, taking into account not only the drug's mechanism of action but also its efficacy and safety profiles in different patient populations, especially in

relation to gender differences. This will help provide a more comprehensive understanding of drug characteristics and better guide clinical drug use.

Secondly, we acknowledge that risk identification of drugs is more important than their classification. Previous studies have conducted risk assessments of AADs for AF based on computer models. However, unlike AADs for ventricular tachycardia, these AADs for AF do not have risk levels suggested by the Comprehensive in vitro Proarrhythmia Assay (CiPA) categorization system, making it possible to develop and validate a mechanistic-based assessment of the proarrhythmic risk of drugs for ventricular tachycardia. Therefore, this study focuses on the role of gender factors in drug screening and personalized medication rather than on drug risk assessment. In future research, we aim to integrate drug efficacy and safety research more comprehensively. We will explore methods to combine the assessment of drug efficacy with the identification of drug risks, especially in the context of gender differences. This could involve developing more accurate risk prediction models that incorporate gender-specific biomarkers and physiological characteristics to better evaluate the overall benefit-risk ratio of drugs and provide more reliable guidance for clinical decision-making.

3. - The use of six different machine learning models (Logistic Regression, SVM, Naive Bayes, XGBoost, Random Forest, K-Nearest Neighbor) represents an application rather than an innovation. These are standard models, and their application here does not constitute a novel methodological contribution. Anyone with access to these models could apply them to a similar dataset to produce predictions.

Respond: We appreciate your comment regarding the methodology of our study. Acknowledging that our method is an existing method, we emphasize that the focus of this paper is on the impact of gender factors on drug efficacy and the underlying mechanisms. By integrating in vitro measurements, in silico models, and machine learning tools in a unique way, we were able to specifically address the problem of gender-specific drug response in atrial fibrillation. This combination of approaches provides a more comprehensive understanding of the complex interactions between drugs, gender, and cardiac electrophysiology. In the revised MS, we have summarized the main contributions of this study and provided a new section **"Comparison with previous modelling studies"**.

Changes in Text (Page 13, Lines 343- 356, Pages 15-16, Lines 411- 432)

Our main findings included: (1) Significant differences in AP and CT biomarkers between virtual male and female patients within the drug population; (2) AP biomarkers were more important than CT biomarkers in drug classification, though CT biomarkers were essential for optimal classification accuracy; (3) ΔAPA and ΔRMP were identified as key features reflecting differences between male- and female-specific classifiers; (4) Sex-specific classifiers outperformed non-sex-specific classifiers, while sex-biased classifiers were inferior to sex-specific classifiers; and (5) The sex-dependent differences in drug response may be partly explained by lower levels of IK1, INa, and Ito in female versus male patients. These results underscore the importance of incorporating sex differences in the evaluation of AAD efficacy and emphasize the potential for personalized medicine approaches in treating AF based on AAD classification. This study indicates the value of combining mechanistic simulations with ML and provides insight into the

role of gender factor in the drug efficacy evaluation.

Our study offers a novel perspective on antiarrhythmic drug classification by introducing gender as a pivotal factor. While there may be some parallels between our methodology and previous studies, our concentrated focus on gender disparities and our integrated approach confer significant value to our research. This study yields fresh insights into the role of gender in drug evaluation and paves the way for more efficacious medical care.

Admittedly, our approach of segregating the data to train two distinct models for different genders might appear straightforward at first glance. However, it represents a targeted response to the extensively documented gender differences in drug response. Our exhaustive analyses of key biomarkers and ionic mechanisms distinguish our work from prior studies. By delving into the disparities between male and female classifiers and pinpointing the specific elements contributing to these differences, we offer a more comprehensive understanding of drug efficacy and potential adverse reactions.

Furthermore, our amalgamation of in vitro measurements, in silico models, and machine learning tools constitutes a unique methodology that presents several advantages over traditional approaches. Previous investigations might have employed one or two of these techniques, but our integrated pipeline provides a more comprehensive and accurate assessment of drug effects. For instance, in silico models enable us to simulate the impacts of drugs on a diverse population of cardiomyocytes. Simultaneously, machine learning tools assist us in identifying patterns and predicting drug efficacy. In vitro measurements serve as a ground truth for our simulations and aid in validating our results.

4. - The reported performance metrics (e.g., SVM accuracy of 0.8095 and specificity of 0.8217) can only tell the model is fitting well on the current features (in distribution cross validation setting), and the binary classification task is not challenging to achieve scores like this, which do not demonstrate significant advancement in predictive capability.

Respond: We appreciate your comment. You are correct that the performance metrics reported may not fully demonstrate a significant advancement in predictive capability, especially considering the binary classification task. Our study's main objective, as emphasized, is to analyze the role of gender factors in drug efficacy evaluation and understand the underlying mechanisms. The classification model was used as a tool to achieve this end. In the revised manuscript, we have summarized the main contributions of this study and further discussed this potential limitation.

Changes in Text (Page 13, Lines 343- 356, Pages 18, Lines 503- 515)

Our main findings included: (1) Significant differences in AP and CT biomarkers between virtual male and female patients within the drug population; (2) AP biomarkers were more important than CT biomarkers in drug classification, though CT biomarkers were essential for optimal classification accuracy; (3) ΔAPA and ΔRMP were identified as key features reflecting differences between male- and female-specific classifiers; (4) Sex-specific classifiers outperformed non-sex-specific classifiers, while sex-biased classifiers were inferior to sex-specific classifiers; and (5) The sex-dependent differences in drug response may be partly explained by lower levels of IK1, INa, and Ito in female versus male patients. These results underscore the importance of incorporating sex differences in the evaluation of AAD efficacy and emphasize the potential for personalized medicine approaches in treating AF based on AAD classification. This study

indicates the value of combining mechanistic simulations with ML and provides insight into the role of gender factor in the drug efficacy evaluation.

Finally, the electrophysiological phenomena of single atrial cells can only partially reflect the reentrant arrhythmias at the organ level in AF. At the organ level, AF may involve gender differences in cardiac anatomical structure, the degree and distribution of fibrosis, and potential organ-level electrical heterogeneity. Therefore, comprehensive gender differences in the evaluation of drug efficacy and safety require more in-depth research. In future investigations, we plan to adopt a more integrated approach that combines single-cell studies with organ-level models. This could involve using in vivo animal models or computational simulations of the whole heart to better understand the complex interactions between drugs, gender differences, and the organ-level electrophysiological environment[68]. By doing so, we hope to obtain a more comprehensive understanding of the relationship between drug efficacy and safety and gender differences at the organ level and translate these findings into more effective clinical strategies.

5. - The reliance on SHAP for feature analysis is standard practice and does not yield novel insights. The identified important features are already well-documented in the literature, and the study does not provide new or surprising findings in this regard.

Respond: Thank you for your comment. This is indeed the case: we identified differences in sex-specific classifiers through SHAP analysis and then through parameter sensitivity analysis, the cardiac electrophysiological mechanisms underlying these differences. Therefore, this is not a method innovation, but a hypothetical analysis.

Changes in Text (Pages 11-13, Lines 293- 331)

The main difference between Male and Female classifiers was in the ranking of ΔRMP and ΔAPA. Compared with feature importance ranking for the male classifier (Figure 9A), ΔRMP is less important than ΔAPA in the feature importance ranking for the female classifier (Figure 9B).

(5) The sex-dependent differences in drug response may be partly explained by lower levels of IK1, INa, and Ito in female versus male patients. These results underscore the importance of incorporating sex differences in the evaluation of AAD efficacy and emphasize the potential for personalized medicine approaches in treating AF based on AAD classification.

1.1.The Different Roles of ΔRMP and ΔAPA in Sex-special Classifiers

To analyze differences in sex-specific classifiers, we performed feature importance analysis using SHAP values. **Figure 9** shows feature importance rankings in sex-specific classifiers. Both male and female classifiers found ΔAPD90 as the most important feature, with AP features (except for APD40) generally more important than CT features. However, excluding CT features substantially decreases the accuracy of the prediction (**Table 3**). *The main difference between Male and Female classifiers was in the ranking of ΔRMP and ΔAPA. Compared with feature importance ranking*

for the male classifier (Figure 9A), ΔRMP is less important than ΔAPA in the feature importance ranking for the female classifier (Figure 9B). Further correlation analysis showed a high negative correlation between ΔRMP and ΔAPA (correlation coefficient of -0.88) (Figure 9C).

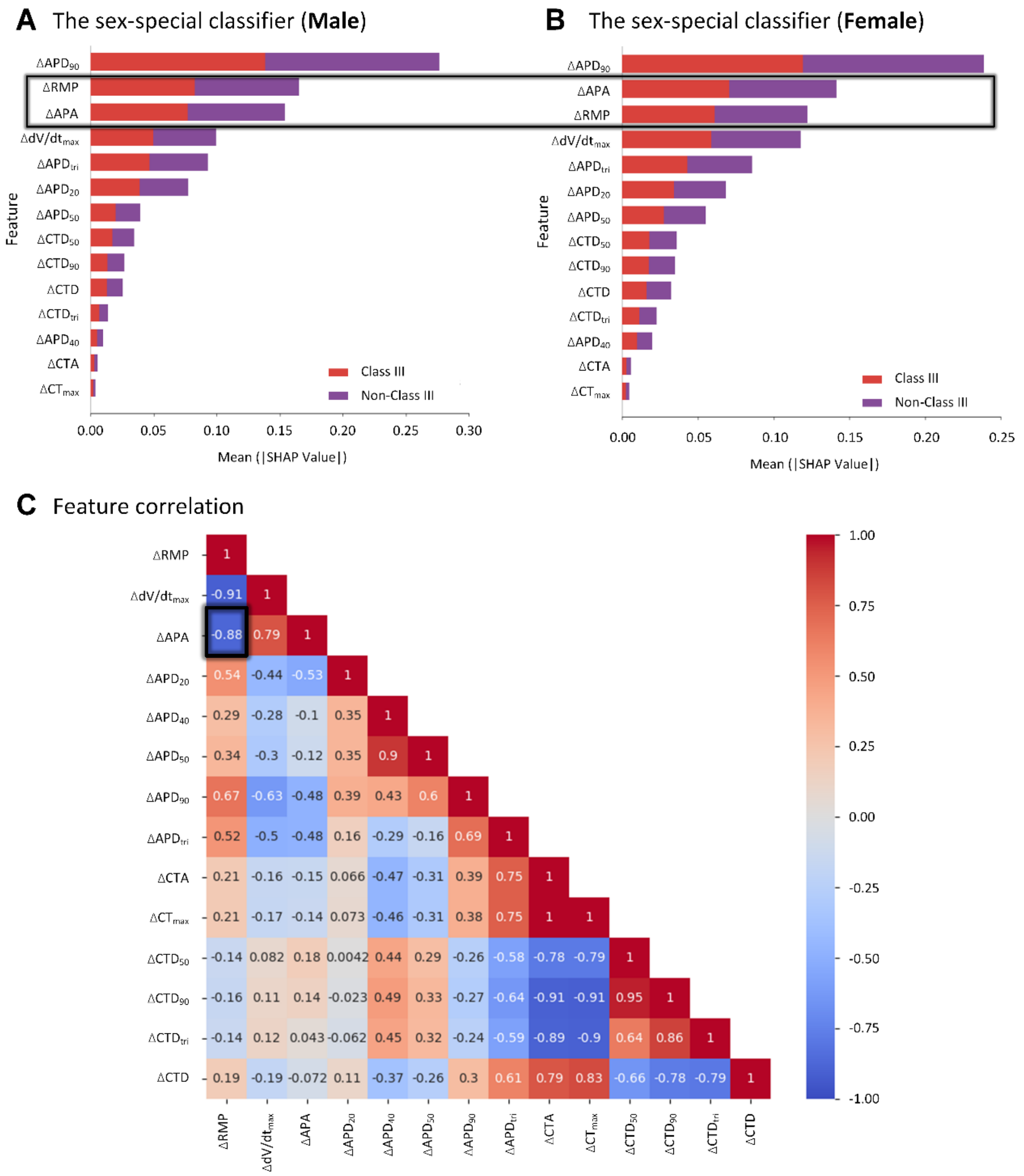

Figure 9. Feature analysis of sex-specific classifiers. Feature importance for the male (A) and female (B) classifiers is measured by the SHAP value. Feature correlation analysis shows that the two key features have a high degree of negative correlation (C).

We also conducted ablation experiments. In these experiments, we removed the feature ΔRMP or ΔAPA to illustrate the impact of these features on classification performance (See ***Table 5****). The experimental results show that ΔRMP and ΔAPA have*

different importance in male and female classifiers. In male classifiers, ΔRMP plays a greater role than ΔAPA, while ΔRMP plays a smaller role than ΔAPA in the female classifier. This provides additional evidence to support our claim about the differences in the ranking of these features between male and female classifiers (See ***Table 6****).*

Table 5. *Feature ablation experimental results.*

Type	features	ACC	SEN	SPE	F1	AUC	MCC
**Sex-special model (Male)**	W/oΔRMP	0.8437 (0.8347,0.8526)	0.7316 (0.7206,0.7427)	0.8823 (0.8609,0.9036)	0.7997 (0.7897,0.8097)	0.8293 (0.8210,0.8376)	0.6815 (0.6620,0.7010)
	W/oΔAPA	0.8542 (0.8477,0.8606)	0.7421 (0.7384,0.7458)	0.8986 (0.8831,0.9142)	0.8128 (0.8059,0.8198)	0.8398 (0.8340,0.8456)	0.7038 (0.6895,0.7182)
	-	0.8802 (0.8751,0.8853)	0.7635 (0.7569,0.7701)	0.9454 (0.9320,0.9587)	0.8450 (0.8389,0.8510)	0.8659 (0.8607,0.8703)	0.7620 (0.7478,0.7762)
**Sex-special model (Female)**	W/oΔRMP	0.8374 (0.8313,0.8434)	0.7485 (0.7379,0.7591)	0.8677 (0.8582,0.8772)	0.8037 (0.7960,0.8113)	0.8285 (0.8223,0.8348)	0.6715 (0.6591,0.6840)
	W/oΔAPA	0.8234 (0.8193,0.8274)	0.7210 (0.7159,0.7262)	0.8594 (0.8490,0.8697)	0.7841 (0.7801,0.7881)	0.8132 (0.8095,0.8169)	0.6437 (0.6348,0.6526)
	-	0.8774 (0.8738,0.8809)	0.7660 (0.7613,0.7707)	0.9484 (0.9442,0.9525)	0.8475 (0.8429,0.8520)	0.8663 (0.8626,0.8699)	0.7601 (0.7509,0.7691)

Table 6. *Role of ΔRMP or ΔAPA in the sex-special models.*

Type	features	ΔACC (%)	ΔSEN (%)	ΔSPE (%)	ΔF1 (%)	ΔAUC (%)	ΔMCC (%)
**Sex-special model (Male)**	**W/oΔRMP**	**3.7%**	**3.2%**	**6.3%**	**4.5%**	**3.7%**	**8.1%**
	W/oΔAPA	2.6%	2.1%	4.7%	3.2%	2.6%	5.8%
**Sex-special model (Female)**	W/oΔRMP	4.0%	1.8%	8.1%	4.4%	3.8%	8.9%
	**W/oΔAPA**	**5.4%**	**4.5%**	**8.9%**	**6.3%**	**5.3%**	**11.6%**

1.2.Lower levels of IK1, INa and Ito in Females Versus Males Explain the Gender Difference in Drug Response

To determine the ionic mechanisms underlying AP differences between male and female AF patients, we identified key ion currents related to RMP and APA via parameter sensitivity analysis and examined statistical differences between male and female AF models. **Figure 10A** shows that RMP is sensitive to GK1 and GNaK, while APA is sensitive to GK1, GNaK, GNa, and Gto. However, there is no sex difference in GNaK but in GK1, GNa, and Gto, with these currents being smaller in females. In addition, there are sex differences in GKs, GKur, GCaL, GNaCa, Grel and Gleak (**Figure 10B**).

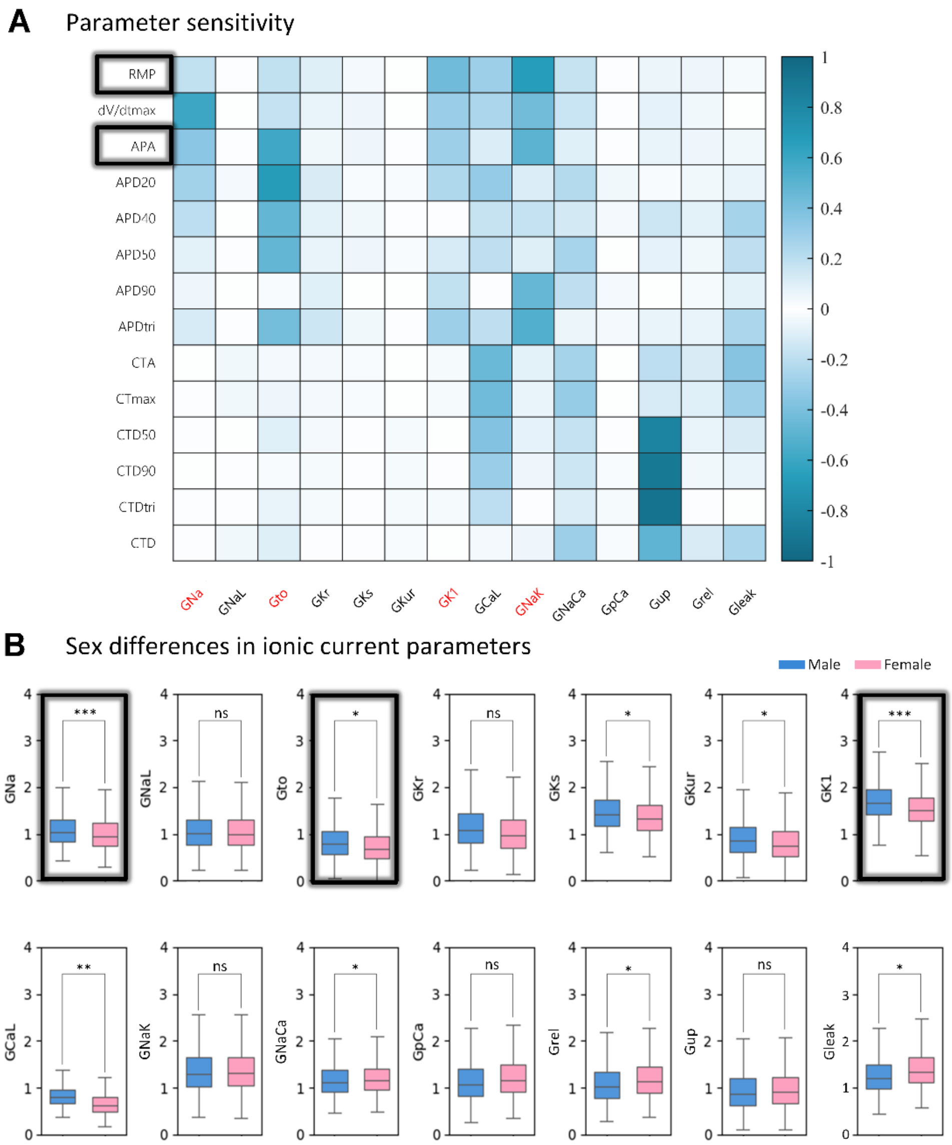

Figure 10. Parameter analysis of sex differences. (A) Sensitivity analysis via ion current parameters (x-axis) versus the model biomarkers (y-axis). (B) Boxplots of ion current parameters in males versus females. The p-values are indicated and the stars *, **, *** indicate a p-value < 0.05, 0.01 and 0.001 respectively. ns indicates not significant.

6. - Overall, the manuscript lacks sufficient innovation or impact to warrant publication in its current form.

Respond: Thanks for your comments. While we admit that there may not be an outstanding innovation in the method itself, as it builds on existing techniques, our study makes a significant contribution in uncovering the mechanism behind the sex differences in drug effects. This is an important aspect that has been overlooked in many previous studies. By focusing on gender factors and their influence on drug efficacy and underlying mechanisms, we provide new insights that can have implications for personalized medicine and drug development.

In the revised MS, we have provided several sections (i.e., *author summary, main contributions, potential applications in clinical practice and Comparison with previous modelling studies*) to

clarify it.

Changes in Text

The challenges posed by the cross-class characteristics of drugs and gender differences in drug response are significant in modern medicine. Traditional methods of drug classification fall short when dealing with drugs that act on multiple targets across different classes. Additionally, male and female patients often respond differently to the same drug due to variations in ion currents, hormonal influences, and calcium handling properties. This necessitates a shift in approach to drug classification and prescribing. In this study, we introduce a pipeline that combines in vitro measurements, in silico models, and machine learning tools to address these challenges. We create a population of calibrated sex-specific models to simulate the effects of drugs on action potential (AP) and calcium transient (CT). By extracting biomarkers from these simulations, we train machine learning classifiers to predict true Class III antiarrhythmic drugs. Our findings show that sex-specific classifiers significantly outperform non-sex-specific classifiers in predicting drug efficacy. We identify key biomarkers that are important for differentiating male and female responses to antiarrhythmic drugs (AADs). Our study highlights the potential of computational models and machine learning in enhancing drug screening processes and developing sex-special treatment strategies. Overall, our work provides new insights into the role of gender factors in drug evaluation and paves the way for more effective and sex-special medical care.

Our main findings included: (1) Significant differences in AP and CT biomarkers between virtual male and female patients within the drug population; (2) AP biomarkers were more important than CT biomarkers in drug classification, though CT biomarkers were essential for optimal classification accuracy; (3) ΔAPA and ΔRMP were identified as key features reflecting differences between male- and female-specific classifiers; (4) Sex-specific classifiers outperformed non-sex-specific classifiers, while sex-biased classifiers were inferior to sex-specific classifiers; and (5) The sex-dependent differences in drug response may be partly explained by lower levels of IK1, INa, and Ito in female versus male patients. These results underscore the importance of incorporating sex differences in the evaluation of AAD efficacy and emphasize the potential for personalized medicine approaches in treating AF based on AAD classification. This study indicates the value of combining mechanistic simulations with ML and provides insight into the role of gender factor in the drug efficacy evaluation.

Our study offers a novel perspective on antiarrhythmic drug classification by introducing gender as a pivotal factor. While there may be some parallels between our methodology and previous studies, our concentrated focus on gender disparities and our integrated approach confer significant value to our research. This study yields fresh insights into the role of gender in drug evaluation and paves the way for more efficacious medical care.

Admittedly, our approach of segregating the data to train two distinct models for different genders might appear straightforward at first glance. However, it represents a targeted response to the extensively documented gender differences in drug response.

Our exhaustive analyses of key biomarkers and ionic mechanisms distinguish our work from prior studies. By delving into the disparities between male and female classifiers and pinpointing the specific elements contributing to these differences, we offer a more comprehensive understanding of drug efficacy and potential adverse reactions.

Furthermore, our amalgamation of in vitro measurements, in silico models, and machine learning tools constitutes a unique methodology that presents several advantages over traditional approaches. Previous investigations might have employed one or two of these techniques, but our integrated pipeline provides a more comprehensive and accurate assessment of drug effects. For instance, in silico models enable us to simulate the impacts of drugs on a diverse population of cardiomyocytes. Simultaneously, machine learning tools assist us in identifying patterns and predicting drug efficacy. In vitro measurements serve as a ground truth for our simulations and aid in validating our results.

This study has practical applications in clinical practice, drug development, and understanding disease mechanisms. By providing a more comprehensive understanding of gender differences in drug response, it can lead to more personalized and effective medical care.

In clinical practice, the ability to accurately predict a drug's efficacy and potential adverse reactions is crucial for prescribing the right medication to each patient[63,64]. The sex-specific classifiers developed in this study can provide valuable insights that go beyond simply looking at the drug's mode of action. By considering gender differences, clinicians can make more informed decisions when prescribing antiarrhythmic drugs. For example, knowing that female patients are more likely to experience certain adverse drug reactions due to differences in ion currents and calcium handling properties[65], doctors can adjust the dosage or choose a different drug altogether. This approach can lead to better patient outcomes, reduced risk of adverse events, and more effective medical care.

The pipeline developed in this study can enhance the drug development and screening process. By accurately classifying drugs based on their effects on different patient populations, researchers can identify potential candidates for further development more efficiently[66]. Additionally, understanding the gender-specific differences in drug response can help in tailoring drug formulations and clinical trials to better meet the needs of different patient groups. This can lead to the development of more targeted and effective drugs, reducing the time and cost of drug development.

Studying the ionic mechanisms underlying gender differences in drug response can contribute to a better understanding of the pathophysiology of atrial fibrillation and other cardiac rhythm disorders[2]. This knowledge can lead to the development of new therapeutic strategies and interventions that take into account gender-specific factors. For example, understanding the role of estrogen in calcium handling properties can help in developing drugs that target these specific pathways, potentially reducing the risk of atrial arrhythmogenesis[67].
