# Supplementary material for: Digital twin for sex-specific identification of class III antiarrhythmic drugs based on in vitro measurements, computer models, and machine learning tools
Source: PLoS Comput Biol. 2025 Jul 3;21(7):e1013154. doi: 10.1371/journal.pcbi.1013154 (PMC12510667; doi:10.1371/journal.pcbi.1013154)
Supplement: Attachment — Submitted filename: PCOMPBIOL-D-24-01299.docx [file pcbi.1013154.s014.docx]

**Summary:** The authors propose an ensemble of ML models that incorporate gender to predict the label of a drug (Class III or non-Class III).

**Clarity:** The paper is generally written. However, I’m not sure if I understand what the machine learning models are trying to predict. Does it try to predict whether a compound is a class III AAD for a given person (different people view the drug as a different class, i.e., individualized class), or for all people (all people view the drug as a predicted class, i.e., general class)? Since the authors mention “personalized treatment” in conclusion, I tend to believe the classifier is predicting drug class for each person. But for the task, it makes more sense to predict general class. If it is to predict individualized class, why would an individual-based classification of a certain drug be useful? Looking at the person’s detailed physiology and drug mechanism is more useful than a coarse class. If it is to predict general class, how is it linked to personalized treatment?

A new section is suggested to formulate the problem, clarifying what the inputs, and outputs, are.

**Significance:** I’m not sure if I get the importance of the work. For either predicting the individualized, or general class, the pharmacist/doctor still need to look at the actual mode of action to determine the use of a drug to a certain patient. The classfication is more for education purpose. If the author believe otherwise, please clarify.

**Originality:** The paper is original by incorporating a new factor into the class prediction: gender. However, the methodology is not very innovative: it merely separates the data to train two different models for genders.

**Scientific Soundness:** The two class label: Class III and Non-Class III are imbalanced (3,1842 for the former and 28,757 for the latter, respectively). However, the chosen classification metrics (e.g., accuracy, sensitivity, specificity etc) are skewed under class imbalance. The authors should provide detailed discussion for the impact of class imbalance and incorporate some unbiased metrics to better evaluate the performance. A useful resource: [1]

Figure2A, 3A, 4A: The color lines are overlapped and it is hard to see if they’re distributed similarly or differently. Maybe think of adjusting the transparency of the lines so the overlaid parts can be seen clearly. Also, the captions say “representative traces” how do the authors ensure the selected lines are representative?

**Conclusion:**

The paper has modest innovation in its methodology. The impact and the scientific soundness should be further elucidated. Hence a major revision is granted.

Ref:

[1] Luque, Amalia, et al. "The impact of class imbalance in classification performance metrics based on the binary confusion matrix." *Pattern Recognition* 91 (2019): 216-231.
