# Supplementary material for: Digital twin for sex-specific identification of class III antiarrhythmic drugs based on in vitro measurements, computer models, and machine learning tools
Source: PLoS Comput Biol. 2025 Jul 3;21(7):e1013154. doi: 10.1371/journal.pcbi.1013154 (PMC12510667; doi:10.1371/journal.pcbi.1013154)
Supplement: Attachment — Submitted filename: Response to Reviewers.docx [file pcbi.1013154.s017.docx]

**Dear Editor-in-Chief,**

I am writing to submit our revised MS entitled "*A Novel Pipeline for Identifying Gender-Specific Class III Antiarrhythmic Drugs Based on In Vitro Measurements, In-Silico Models and Machine Learning Tools*" (Manuscript ID PCOMPBIOL-D-24-01299) for consideration for publication in PLOS Computational Biology.

We thank all Reviewers and editors for their overall positive evaluation of our study and helpful criticisms and suggestions. We believe the revisions made in all three main sections (structure, description, and discussion) as per the editor’s and reviewers’ suggestions have significantly improved our manuscript and clarified our data presentation. To expedite the review, we present our revisions to the manuscript in blue font.

We hope that our detailed response and text revision are sufficient for acceptance of our MS in PLOS Computational Biology.

Best regards,

|   Prof. Alexander V. Panfilov  Department of Physics and Astronomy Gent University, Krijgslaan 281, S9, 9000 Gent  Telephone: +32-9-2644964 Fax: +32-9-2644989  Email: Alexander.Panfilov@UGent.be  http://www.heart.ugent.be/ |   Jichao Zhao, Ph.D., FHRS,  Senior research fellow, Auckland Bioengineering Institute, The University of Auckland, Auckland, New Zealand  Phone: +64 9 923 6505;  Email: j.zhao@auckland.ac.nz  https://unidirectory.auckland.ac.nz/profile/jzha319 |
| --- | --- |

Comments to the Authors:

Please note here if the review is uploaded as an attachment.

Reviewer #1: Thank you for incorporating my feedback, and for the clarifications. The addition of authors summary and further changes in the texts have clarified the significance and originality. The change of evaluation metrics also strengthens the scientific soundness.

**Response:** Thank you very much.

Reviewer #2: Thank you for your detailed and thoughtful responses addressing my previous concerns. I have no further comments and am fully satisfied with the work.

**Response:** Thank you very much.

Reviewer #3: Thank you for the responses and the revisions. After reviewing the responses, I still feel the manuscript falls short of addressing the major concerns.

**Response:** We truly appreciate the reviewer's in-depth comments and the opportunity to further clarify our work.

1. The authors acknowledge that the methodology isn’t novel, and while I appreciate the focus on gender-specific drug responses, the approach is quite standard. Combining in vitro measurements, in silico simulations, and machine learning is useful, but it’s not particularly unique.

**Response:** We sincerely appreciate your thoughtful feedback. We understand your concern regarding the novelty of our approach. You are correct that the combination of in vitro, in silico, and machine learning techniques has been employed in prior studies, particularly in the context of computational analysis of gender differences in antiarrhythmic drug responses. To address your comment, we have revised the manuscript title, replacing the term novel with computational to better reflect the nature of our study. On a broader note, gender differences in antiarrhythmic drug response remain a crucial and, in our view, largely underexplored area of research. In our work, we not only identified these gender differences but also investigated the underlying ionic mechanisms in depth. We believe these insights could have significant implications for advancing personalized medicine. Another key aspect of our study is the creation of a large and diverse set of models—11,847 in total—calibrated using real experimental data. This extensive model set enhances the accuracy and reliability of our predictions, which is essential for robust computational analysis. We hope that these revisions address your concerns and underscore the value of our work. Thank you once again for your constructive comments and for helping us improve the manuscript.

**Changes in the context:**

*A Computational Pipeline for Identifying Gender-Specific Class III Antiarrhythmic Drugs Based on In Vitro Measurements, In-Silico Models and Machine Learning Tools*

*Our study introduces a computational pipeline that integrates in vitro measurements, in silico models, and machine learning (ML) to identify gender-specific Class III AADs.*

*In this study, we introduce a computational pipeline that combines in vitro measurements, in silico models, and machine learning tools to address these challenges.*

*Main aim of this study is to develop a computational pipeline which can be used for in-silico sex-specific drug testing.*

*In this study, we developed a computational pipeline that combines mechanistic modeling with ML analyses, and we applied this to examine individualized drug response. Mechanistic simulations were used to generate an experimentally-calibrated population of virtual atrial cells representing AF patients and the corresponding drug population of thousands of cardiomyocytes representing AF patients after taking medication.*

*Our study offers a perspective on antiarrhythmic drug classification by introducing gender as a pivotal factor. While there may be some parallels between our methodology and previous studies, our concentrated focus on gender disparities and our integrated approach confers significant value to our research. This study yields fresh insights into the role of gender in drug evaluation and paves the way for more efficacious medical care.*

*Furthermore, our amalgamation of in vitro measurements, in silico models, and machine learning tools constitutes a methodology that presents several advantages over traditional approaches. Previous investigations might have employed one or two of these techniques, but our integrated pipeline provides a more comprehensive and accurate assessment of drug effects. For instance, in silico models enable us to simulate the impacts of drugs on a diverse population of cardiomyocytes. Simultaneously, machine learning tools assist us in identifying patterns and predicting drug efficacy. In vitro measurements serve as a ground truth for our simulations and aid in validating our results.*

2. The issue of relying solely on previously known biomarkers still stands. While I understand that discovering new biomarkers wasn’t the goal, the study doesn’t offer any fresh insights about the ones used. Simply reiterating their relevance in a gender-specific context feels more like repackaging than advancing the field.

**Response:** We are truly grateful for your insightful observation. You are correct that our study did not focus on the discovery of new biomarkers. We fully agree that the introduction of novel biomarkers is an extremely important area of research, as highlighted in numerous studies and grant proposals. However, most current modeling studies in this field rely on accepted and validated biomarkers. As emphasized in your previous comment, the primary aim of our study is the application of these established biomarkers to an important problem, with a particular focus on gender-specificity. In our work, we demonstrated that all 14 biomarkers included in our study contribute to achieving optimal classification accuracy, with certain biomarkers, such as ΔAPA and ΔRMP, showing relatively greater significance. This finding challenges the common approach of focusing on only a few well-established biomarkers and highlights the need for a more comprehensive and inclusive strategy. Furthermore, we identified differences in biomarker importance between male and female classifiers. Specifically, ΔRMP and ΔAPA play distinct roles in sex-specific classifications. This observation is particularly significant and should be considered in future studies exploring gender differences in drug response.

**Changes in the context:**

*Our study did not center around the discovery of new biomarkers, however, it enriches the comprehension of the functions of pre-existing biomarkers within the framework of gender-specific drug responses. We have demonstrated that, among the 14 biomarkers under consideration, each plays a crucial part in attaining optimal classification accuracy, with certain ones such as ΔAPA and ΔRMP being of relatively greater significance.*

*The ML analyses indicated that the gender factor needs to be considered in drug efficacy assessments.* *Our main findings included: (1) Significant differences in AP and CT biomarkers between virtual male and female patients within the drug population; (2) AP biomarkers were more important than CT biomarkers in drug classification, though CT biomarkers were essential for optimal classification accuracy; (3) ΔAPA and ΔRMP were identified as key features reflecting differences between male- and female-specific classifiers; (4) Sex-specific classifiers outperformed non-sex-specific classifiers, while sex-biased classifiers were inferior to sex-specific classifiers; and* *(5) The sex-dependent differences in drug response may be partly explained by lower levels of IK1, INa, and Ito in female versus male patients. These results underscore the importance of incorporating sex differences in the evaluation of AAD efficacy and emphasize the potential for personalized medicine approaches in treating AF based on AAD classification. This study indicates the value of combining mechanistic simulations with ML and provides insight into the role of gender factor in the drug efficacy evaluation.*

3. The binary classification task is very limiting. While I get that the authors wanted to focus on Class III and Class I drugs, this approach feels too narrow, especially when more nuanced classifications could have added real value. Acknowledging this as a limitation and suggesting future directions doesn’t solve the problem in the current study.

**Response:** We would like to sincerely thank you for your insightful comment. While we acknowledge that binary classification is indeed restrictive, it is also straightforward. For this reason, we chose it as an essential initial step, given the prominent role of Class III drugs in the treatment of atrial fibrillation. Our goal was to establish a fundamental proof of concept and develop a methodological framework that could serve as a foundation for future research. We recognize the limitations of this approach and have addressed them in the manuscript by outlining potential future directions. These include expanding the classification framework to encompass a broader range of drug categories and adopting more refined criteria. By doing so, we aim to build upon the groundwork we have carefully laid and gain a deeper and more comprehensive understanding of drug characteristics across diverse patient populations. We greatly value your feedback and are committed to advancing our research in alignment with these considerations. Thank you once again for your valuable input.

**Changes in the context:**

*The current study's binary classification task, which divided drugs into Class III and non-Class III (while these drugs can be further categorized into more than four according to the Vaughan Williams classification system), was rather limiting. The focus on Class III drugs was due to their extensive use in AF and potential risk of inducing TdP, a dangerous form of polymorphic ventricular tachycardia. Our intention was to perform a fundamental proof of concept using as the first step the most straightforward (binary) classification and formulate a methodological framework that could serve as a springboard for subsequent investigations*. *From a clinical application perspective, further research is required to explore more detailed classification methods. Future work should expand the drug classification scope to include a more diverse range of drug categories and develop more refined criteria. This should consider not only the drug's mechanism of action but also its efficacy and safety in different patient populations, especially in relation to gender differences.*

4. It also validates the point that the machine learning application here is more about routine analysis than a novel contribution.

**Response:** Thank you very much for your assessment. You are correct that the machine learning algorithms we employed are commonly used. However, in the revised version of the manuscript, we no longer claim that our approach is novel, but instead focus on the results achieved using this methodology. For the first time, we demonstrate that when applied to gender-specific problems, our sex-specific classifiers, such as SVM, achieve over 87% accuracy. This provides clear evidence of the effectiveness of our approach. Furthermore, it highlights the potential of this methodology for drug screening procedures and advancing personalized medicine. We hope this explanation clarifies the unique aspects of our machine learning application in this study. Thank you once again for your valuable feedback.

**Changes in the context:**

*In clinical practice, the ability to accurately predict a drug's efficacy and potential adverse reactions is crucial for prescribing the right medication to each patient63,64. The sex-specific classifiers developed in this study can provide valuable insights that go beyond simply looking at the drug's mode of action. By considering gender differences, clinicians can make more informed decisions when prescribing antiarrhythmic drugs. For example, knowing that female patients are more likely to experience certain adverse drug reactions due to differences in ion currents and calcium handling properties65, doctors can adjust the dosage or choose a different drug altogether. This approach can lead to better patient outcomes, reduced risk of adverse events, and more effective medical care.*

*The pipeline developed in this study can enhance the drug development and screening process. By accurately classifying drugs based on their effects on different patient populations, researchers can identify potential candidates for further development more efficiently66. Additionally, understanding the gender-specific differences in drug response can help in tailoring drug formulations and clinical trials to better meet the needs of different patient groups. This can lead to the development of more targeted and effective drugs, reducing the time and cost of drug development.*

*Admittedly, our approach of segregating the data to train two distinct models for different genders might appear straightforward at first glance. However, it represents a targeted response to the extensively documented gender differences in drug response. Our exhaustive analyses of key biomarkers and ionic mechanisms distinguish our work from prior studies. By delving into the disparities between male and female classifiers and pinpointing the specific elements contributing to these differences, we offer a more comprehensive understanding of drug efficacy and potential adverse reactions.*

*Furthermore, our amalgamation of in vitro measurements, in silico models, and machine learning tools constitutes a methodology that presents several advantages over traditional approaches. Previous investigations might have employed one or two of these techniques, but our integrated pipeline provides a more comprehensive and accurate assessment of drug effects. For instance, in silico models enable us to simulate the impacts of drugs on a diverse population of cardiomyocytes. Simultaneously, machine learning tools assist us in identifying patterns and predicting drug efficacy. In vitro measurements serve as a ground truth for our simulations and aid in validating our results.*

5. The authors themselves confirm that this is more of a hypothetical analysis rather than methodological innovation. While SHAP and sensitivity analysis are fine tools, they’re standard, and the study doesn’t bring anything particularly new to the table.

**Response:** Thank you very much for your remarks. In light of your comments, we no longer claim that our study represents a methodological innovation. However, our work does introduce an application of these methodologies to unique data, addressing a practically important problem and yielding interesting new findings on the significance of various biomarkers in gender-specific contexts. Our approach combines experimental data with specially designed computational models. These models were calibrated using real patient data, followed by comprehensive simulations of drug effects and subsequent classification of the results. In our study, SHAP and sensitivity analysis were strategically employed to gain deeper insights into the mechanisms underlying gender differences in drug response. For example, sensitivity analysis allowed us to pinpoint specific ion currents responsible for the disparities in action potentials observed between male and female patients. We believe this comprehensive approach offers valuable contributions for researchers interested in gender-specific differences in antiarrhythmic drug action and personalized antiarrhythmic therapies. Thank you once again for your thoughtful input.

**Changes in the context:**

*(2) AP biomarkers were more important than CT biomarkers in drug classification, though CT biomarkers were essential for optimal classification accuracy; (3) ΔAPA and ΔRMP were identified as key features reflecting differences between male- and female-specific classifiers; (4) Sex-specific classifiers outperformed non-sex-specific classifiers, while sex-biased classifiers were inferior to sex-specific classifiers; and (5) The sex-dependent differences in drug response may be partly explained by lower levels of IK1, INa, and Ito in female versus male patients. These results underscore the importance of incorporating sex differences in the evaluation of AAD efficacy and emphasize the potential for personalized medicine approaches in treating AF based on AAD classification.*

We hope these clarifications effectively address the reviewer's concerns and underscore the unique contributions of our study. We remain dedicated to further advancing this research and greatly appreciate the reviewer’s valuable feedback in shaping our future efforts.
