# Supplementary material for: Digital twin for sex-specific identification of class III antiarrhythmic drugs based on in vitro measurements, computer models, and machine learning tools
Source: PLoS Comput Biol. 2025 Jul 3;21(7):e1013154. doi: 10.1371/journal.pcbi.1013154 (PMC12510667; doi:10.1371/journal.pcbi.1013154)
Supplement: Attachment — Submitted filename: Response_to_Reviewers_auresp_3.docx [file pcbi.1013154.s018.docx]

Professor Yang Lu, Ph.D., Academic Editor

Professor Jason Haugh, Section Editor

PLOS Computational Biology

**Dear Editor-in-Chief,**

I am writing to submit our revised MS entitled "*Sex-specific Identification of Class III Antiarrhythmic Drugs Based on In Vitro Measurements, Computer models, and Machine Learning Tools*" (Manuscript ID PCOMPBIOL-D-24-01299R2) for consideration for publication in *PLOS Computational Biology*.

We thank all Reviewers and editors for their overall positive evaluation of our study and helpful criticisms and suggestions. We believe the revisions made in all three main sections (structure, description, and discussion) as per the editor’s and reviewers’ suggestions have significantly improved our manuscript and clarified our data presentation. To expedite the review, we present our revisions to the manuscript in blue font. We have made the following significant changes to improve the paper:

1. Regarding concerns of Reviewer #4 about expanding the analysis to include other influencing factors to make the study more impactful, we have thoroughly addressed these points by (1) clarifying how our model inherently accounts for multiple clinical factors through experimental data calibration, (2) expanding discussions on the physiological relevance of these factors, and (3) explicitly outlining methodological trade-offs and future directions. In the revised manuscript, we updated the **Methods Section (Page 7, Lines 158-165)**, the **Discussion Section (Page 18, Lines 425-433)** and the **Limitations Section (Page 21, Lines 514-525)*.***
2. Regarding the Reviewer #4’s suggestion for biomarker discovery, we have leveraged SHAP interpretability analysis to propose potential biomarker candidates, aligning with the methodological focus of the paper. In the revised manuscript, we identified the integrated action potential area (AREA), a novel composite metric combining APA, APD, and RMP, as a key electrophysiological feature. This biomarker ranked as the fourth most important predictor in our model and improved classification accuracy by ~2%, providing a testable hypothesis for future experimental validation. In the revised manuscript, we added the **Section 2.6 (Page 14, Lines 318–326), a New Table 7 and a New Supplementary Figure (S0 Text).**

We hope that our detailed response and text revision are sufficient for acceptance of our MS in PLOS Computational Biology.

Thank you very much for your consideration,

Best regards,

|   Prof. Alexander V. Panfilov  Department of Physics and Astronomy Gent University, Krijgslaan 281, S9, 9000 Gent  Telephone: +32-9-2644964 Fax: +32-9-2644989  Email: Alexander.Panfilov@UGent.be  http://www.heart.ugent.be/ |   Jichao Zhao, Ph.D., FHRS,  Senior research fellow, Auckland Bioengineering Institute, The University of Auckland, Auckland, New Zealand  Phone: +64 9 923 6505;  Email: j.zhao@auckland.ac.nz  https://unidirectory.auckland.ac.nz/profile/jzha319 |
| --- | --- |

**Journal Requirements:**

1) Please amend your detailed Financial Disclosure statement. This is published with the article. It must therefore be completed in full sentences and contain the exact wording you wish to be published.

State the initials, alongside each funding source, of each author to receive each grant. For example: "This work was supported by the National Institutes of Health (####### to AM; ###### to CJ) and the National Science Foundation (###### to AM)."

2) Please ensure that the funders and grant numbers match between the Financial Disclosure field and the Funding Information tab in your submission form. Note that the funders must be provided in the same order in both places as well. Currently, the order of this grant "075-15-2022-304" is different in both places. In addition, this information "China Scholarship Council under Project No. 202206785002" is missing from the Funding Information tab.

Please indicate by return email the full and correct funding information for your study and confirm the order in which funding contributions should appear.

**Response:** We sincerely appreciate the editor’s careful guidance. We have amended the Financial Disclosure section as requested, now stating funding sources in full sentences with exact wording for publication.

**Changes in the context:**

*This work was supported by the Natural Science Foundation of Guangdong Province (2023A1515012833 to J.B.; 2024A1515011886 to J.B), the National Natural Science Foundation of China (61901192 to J.B.), High-End Foreign Expert Recruitment Program (H20240205 to J.B.), China Scholarship Council (202206785002 to J.B.), and the Ministry of Science and Higher Education of the Russian Federation within the framework of state support for the creation and development of World-Class Research Centers “Digital Biodesign and Personalized Healthcare”(075-15-2022-304 to A.P.). The funders had no role in study design, data collection and analysis, decision to publish, or preparation of the manuscript.*

**Reviewers' comments:**

Reviewer's Responses to Questions

Reviewer #1: The authors have addressed my concerns in the previous revision already. In the current revision, the authors are thus trying to clarifying the concerns the third reviewers have. The concerns are mainly about the originality and the limited task scope. The authors have revised the manuscript and title to no longer claiming the approach is novel but instead emphasize on its application to unique data, addressing a practically important problem and yielding interesting new findings in gender-specific context. Personally I have no further questions for the authors' responses.

**Response:** Thank you very much.

Reviewer #2: Thank you for your responses. While the paper has certain limitations in terms of methodological innovation, the reviewer recognizes its value. The authors have generated a substantial amount of simulated data and conducted a rigorous analysis of biomarkers and the effects of class III drugs on atrial fibrillation (AF) patients in a gender-specific context. Additionally, the work highlights several intriguing directions for future research, including one with clinical potential for gender-based drug prescription. The results are sufficiently robust to warrant publication in their current form.

**Response:** Thank you very much.

Reviewer #4: The paper presents a pipeline for identifying gender-specific Class III antiarrhythmic drugs using in vitro data, in silico models, and ML techniques. The sex-specific classifiers achieve high accuracy and provide interpretable biomarker insights, aligning with the goals of precision medicine. However, the study's novelty, particularly as a methods-focused paper, is insufficient and requires significant improvement.

1. I agree with the third reviewer that the paper's novelty is limited. PLOS Computational Biology emphasizes methodological innovation, and this study does not introduce a sufficiently novel method. The only notable aspect is the consideration of gender in classification. Expanding the analysis to include other influencing factors, such as age, weight, or gender-specific characteristics in other drugs or diseases, would make the study more impactful. Presenting only a pipeline is too limited.

**Response:** We sincerely appreciate the reviewer’s constructive feedback on enhancing the study’s impact by considering additional influencing factors. We apologize for any lack of clarity in our original manuscript regarding this aspect. In our study, the computational model was constructed and calibrated using experimental data (S Pecha et al., 2023) that inherently capture the integrated effects of diverse clinical factors (e.g., age, BMI, hypertension, and coronary artery disease) on cardiomyocyte electrophysiology. Explicitly incorporating each factor—along with their interactions—would introduce substantial model complexity and pose challenges for rigorous validation, given the limitations of available datasets. Thus, our current approach prioritizes a balance between biological comprehensiveness and computational feasibility.

To address these concerns, we have made the following revisions:

- **In the Methods Section,** we have clarified how our model inherently reflects the influence of clinical factors (e.g., age, sex, BMI) through its calibration to experimental data, which captures their integrated effects on cardiomyocyte electrophysiology.
- **In the Discussion Section,** we expanded our discussion to explicitly examine how these factors influence human atrial electrophysiology, drawing upon prior studies to contextualize our findings. This addition underscores the broader physiological relevance of our work.
- **In the Limitations Section,** we added a dedicated discussion acknowledging the trade-offs in our methodology, particularly the challenges of explicitly modeling individual factors and their interactions given current data constraints. We also outline a forward-looking perspective on how future studies could incorporate these factors as more comprehensive datasets become available.

*Pecha S, Ismaili D, Geelhoed B, et al. Resting membrane potential is less negative in trabeculae from right atrial appendages of women, but action potential duration does not shorten with age. J Mol Cell Cardiol. 2023;176:1-10.*

**Changes in the context:**

*Page 7, Lines 158-165: We generated a large initial population of 100,000 models by randomly varying key parameters related to human atrial electrophysiology. This population was calibrated to retain only those models that were fully consistent with the experimentally observed ranges of seven biomarkers that inherently capture the integrated effects of diverse clinical factors (e.g., age, sex, body mass index, hypertension, left ventricular hypertrophy, AF and beta-blocker usage) on human atrial electrophysiology ^58^. The calibration process reduced the initial population to 11,847 accepted models (5,663 males and 6,184 females) (****See Method 5.3****).*

*Page 18, Lines 425-433: Unlike previous modeling studies that relied on idealized systems or animal-derived parameters74,75, our human-centric framework is uniquely grounded in patient-specific electrophysiological data that intrinsically captures integrated clinical variables (age, comorbidities, polypharmacy)58. This human-data-driven approach reveals fundamental sex-specific electrophysiological phenotypes: female cardiomyocytes exhibit a less negative RMP and demonstrate attenuated age-related APD90. These findings enable us to model sex as a biological variable with mechanistic underpinnings rather than merely as a statistical covariate, providing novel insights into sex-divergent pharmacodynamics and paving the way for precision medicine strategies.*

*Page 21, Lines 514-525: Thirdly, while AF pathophysiology critically involves calcium dysregulation and action potential shortening, the experimental data used to calibrate our computational model—though capturing integrated effects of clinical factors (age, BMI, hypertension, etc.) on cardiomyocyte electrophysiology—lacked explicit calcium transient measurements58. This precluded implementation of biochemically detailed calcium-handling systems and obscured potential gender differences in calcium dynamics (e.g., sex hormone modulation of SERCA/RyR2 activity). While population-level calibration implicitly accounts for comorbidities, we could not resolve how calcium dysregulation interacts with clinical variables (e.g., beta-blocker use) across sexes. Future studies will employ sex-stratified iPSC-CM calcium imaging and mechanistic modeling (e.g., CaMKII/RyR2 phosphorylation states) to bridge this gap, enabling more precise evaluation of drug-gender interactions in AF.*

2. Regarding the third reviewer’s suggestion for new biomarker discovery, the authors might not need to fully identify new biomarkers but could instead propose potential candidates using SHAP interpretability analysis. This would sufficiently address the requirement in a method-focused paper.

**Response:** We sincerely appreciate the reviewer's thoughtful suggestion regarding biomarker discovery. Following this recommendation, we conducted comprehensive SHAP analysis to identify potential biomarker candidates in our computational framework. Through this interpretable machine learning approach, we discovered that the integrated action potential area (a novel composite metric combining APA, APD, and RMP parameters) emerged as a significant electrophysiological feature, ranking as the fourth most important predictor in our model. The inclusion of this biomarker candidate improved our model's classification accuracy by ~2%. This finding provides a testable hypothesis for future experimental validation studies. We have added these new analyses in Section 2.6 and presented the results in Table 7 and Figure of S0 Text.

**Changes in the context:**

*Page 14, Lines 318-326: Feature importance analysis revealed that ΔRMP, ΔAPA, and ΔAPD90 were the three most critical features. Interestingly, the combination of RMP, APA, and APD90 effectively represents the area under the action potential curve (AREA). Therefore, we extracted this composite feature (ΔAREA) and investigated its effects on classification performance and its role in the predictive model. Experimental results demonstrated that incorporating this new biomarker led to a consistent improvement by ~2% in the model's AUC (See* ***Table 7****). Furthermore, subsequent feature importance analysis ranked this newly derived biomarker as the fourth most influential in the classification process (****Figure of S0 Text****).*

*New Table 7.* **Effects of the new geometric biomarker on classification performance**.

| **Type** | **Models** | **ACC** | **SEN** | **SPE** | **F1** | **MCC** | **AUC** |
| --- | --- | --- | --- | --- | --- | --- | --- |
| **Sex-special model (Male)** | LR | 0.7471  (0.7412,0.7529) | 0.6849  (0.6683,0.7014) | 0.7122  (0.7084,0.7159) | 0.6982  (0.6881,0.7082) | 0.4810  (0.4680,0.4940) | 0.73922  (0.7320,0.7463) |
|  | SVM | **0.8922**  **(0.8878,0.8967)** | 0.8551  (0.8405,0.8698) | **0.8886**  **(0.8848,0.8924)** | **0.8715**  **(0.8650,0.8779)** | **0.7794**  **(0.7701,0.7886)** | **0.8875**  **(0.8819,0.8932)** |
|  | NB | 0.6316  (0.6249,0.6384) | **0.9404**  **(0.9324,0.9484)** | 0.5396  (0.5350,0.5442) | 0.6857  (0.6801,0.6913) | 0.3877  (0.3725,0.4028) | 0.6708  (0.6640,0.6776) |
|  | XGB | 0.8847  (0.8788,0.8907) | 0.8499  (0.8374,0.8623) | 0.8767  (0.8710,0.8824) | 0.8630  (0.8554,0.87075) | 0.7640  (0.7517,0.7762) | 0.8803  (0.8737,0.8869) |
|  | RF | 0.8660  (0.8583,0.8737) | 0.8317  (0.8190,0.8443) | 0.8515  (0.8399,0.8632) | 0.8414  (0.8322,0.8506) | 0.7257  (0.7100,0.7415) | 0.8616  (0.8537,0.8695) |
|  | KNN | 0.8504  (0.8460,0.8547) | 0.8179  (0.8049,0.8309) | 0.8297  (0.8232,0.8362) | 0.8237  (0.8175,0.8298) | 0.6940  (0.6847,0.7032) | 0.8463  (0.8411,0.8514) |
| **Sex-special model (Female)** | LR | 0.7262  (0.7195,0.7330) | 0.6833  (0.6749,0.6917) | 0.6995  (0.6903,0.7087) | 0.6913  (0.6843,0.6983) | 0.4456  (0.4323,0.4590) | 0.7222  (0.7157,0.7288) |
|  | SVM | **0.8905**  **(0.8866,0.8943)** | 0.8535  (0.8462,0.8609) | **0.8972**  **(0.8942,0.9003)** | **0.8748**  **(0.8701,0.8796)** | **0.7784**  **(0.7705,0.7862)** | **0.8870**  **(0.8828,0.8912)** |
|  | NB | 0.6207  (0.6125,0.6288) | **0.9168**  **(0.9115,0.9221)** | 0.5459  (0.5404,0.5515) | 0.6844  (0.6786,0.6902) | 0.3422  (0.3257,0.3587) | 0.6483  (0.6405,0.6562) |
|  | XGB | 0.8772  (0.8745,0.8798) | 0.8557  (0.8520,0.8594) | 0.8685  (0.8644,0.8726) | 0.8621  (0.8591,0.8650) | 0.7515  (0.7461,0.7569) | 0.8752  (0.8725,0.8778) |
|  | RF | 0.8598  (0.8541,0.8654) | 0.8326  (0.8243,0.8408) | 0.8515  (0.8443,0.8587) | 0.8419  (0.8354,0.8484) | 0.7162  (0.7046,0.7277) | 0.8572  (0.8514,0.8630) |
|  | KNN | 0.8447  (0.8387,0.8507) | 0.8130  (0.7997,0.8263) | 0.8363  (0.8301,0.8425) | 0.8244  (0.8167,0.8322) | 0.6857  (0.6733,0.6980) | 0.8418  (0.8352,0.8483) |
| **Non-sex-special model** | LR | 0.6774  (0.6731,0.6817) | 0.8479  (0.8415,0.8544) | 0.6562  (0.6528,0.6597) | 0.7397  (0.7360,0.7433) | 0.3441  (0.3347,0.3534) | 0.6642  (0.6598,0.6686) |
|  | SVM | **0.8316**  **(0.8292,0.8341)** | 0.8554  (0.8470,0.8638) | **0.8347**  **(0.8275,0.8419)** | **0.8448**  **(0.8428,0.8469)** | **0.6613**  **(0.6563,0.6663)** | **0.8298**  **(0.8270,0.8327)** |
|  | NB | 0.6565  (0.6519,0.6611) | **0.9041**  **(0.8975,0.9108)** | 0.6238  (0.6210,0.6266) | 0.7382  (0.7344,0.7421) | 0.3290  (0.3174,0.3406) | 0.6373  (0.6328,0.6419) |
|  | XGB | 0.8287  (0.8253,0.8321) | 0.8476  (0.8418,0.8533) | 0.8349  (0.8306,0.8392) | 0.8412  (0.8379,0.8444) | 0.6554  (0.6486,0.6622) | 0.8272  (0.8238,0.8306) |
|  | RF | 0.8050  (0.8000,0.8101) | 0.8237  (0.8116,0.8357) | 0.8146  (0.8110,0.8182) | 0.8190  (0.8132,0.8249) | 0.6079  (0.5979,0.6179) | 0.8036  (0.7989,0.8083) |
|  | KNN | 0.7992  (0.7949,0.8036) | 0.8539  (0.8464,0.8614) | 0.7890  (0.7814,0.7965) | 0.8201  (0.8168,0.8234) | 0.5964  (0.5878,0.6049) | 0.7950  (0.7902,0.7998) |

*New Figure.* *Feature analysis of sex-specific classifiers. Feature importance for the male (A) and female (B) classifiers is measured by the SHAP value. Feature correlation analysis shows that the two key features have a high degree of negative correlation (C).*

3. Given the lack of novelty, the authors should consider incorporating more nuanced classifications as proposed by third reviewer or collecting additional datasets.

**Response:** We would like to sincerely thank you for your insightful comment. While we acknowledge that binary classification is indeed restrictive, it is also straightforward. For this reason, we chose it as an essential initial step, given the prominent role of Class III drugs in the treatment of atrial fibrillation. Our goal was to establish a fundamental proof of concept and identify gender-specific Class III AADs by integrating in vitro measurements, in silico models, and machine learning (ML). We recognize the limitations of this approach and have addressed them in the manuscript by outlining potential future directions. These include expanding the classification framework to encompass a broader range of drug categories and adopting more refined criteria. By doing so, we aim to build upon the groundwork we have carefully laid and gain a deeper and more comprehensive understanding of drug characteristics across diverse patient populations. We greatly value your feedback and are committed to advancing our research in alignment with these considerations. Thank you once again for your valuable input.

**Changes in the context:**

*Page 20, Lines 487-499: Firstly, the current study's binary classification task, which divided drugs into Class III and non-Class III (while these drugs can be further categorized into more than four according to the Vaughan Williams classification system), was rather limiting. The focus on Class III drugs was due to their extensive use in AF and potential risk of inducing TdP, a dangerous form of polymorphic ventricular tachycardia. Our intention was to perform a fundamental proof of concept using as the first step the most straightforward (binary) classification and formulate a methodological framework that could serve as a springboard for subsequent investigations. From a clinical application perspective, further research is required to explore more detailed classification methods. Future work should expand the drug classification scope to include a more diverse range of drug categories and develop more refined criteria. This should consider not only the drug's mechanism of action but also its efficacy and safety in different patient populations, especially in relation to gender differences.*

4. If the paper aims to function as a pipeline, the authors should improve their GitHub repository by providing clear instructions and examples for users and publish a package which can make it transfer to other tasks, making it more accessible and practical for the research community.

**Response:** We sincerely thank the reviewer for this valuable suggestion to improve the accessibility of our computational pipeline. In response, we have significantly enhanced our GitHub repository (https://github.com/xuanyuanzaishui/Sex-Specific-Classification-of-Antiarrhythmic-Drugs) by providing detailed documentation, step-by-step tutorials, and example datasets. These improvements make the pipeline readily transferable to new classification tasks while maintaining reproducibility of our published results. We believe these enhancements will greatly increase the utility and impact of our work within the research community.

**Changes in the context:** *Modeling and simulations were conducted on desktop servers. The simulations used to create the population in the project were implemented using the stiff ordinary differential equation solver ode15s in MATLAB 2022b. The ML models were implemented in Python, using the packages NumPy, Pandas, Scikit-Learn, SHAP, and Pickle. The source code used in this study can be downloaded from the GitHub website (*[*https://github.com/xuanyuanzaishui/Sex-Specific-Classification-of-Antiarrhythmic-Drugs*](https://github.com/xuanyuanzaishui/Sex-Specific-Classification-of-Antiarrhythmic-Drugs)*).*

We hope these clarifications effectively address the reviewer's concerns and underscore the unique contributions of our study. We remain dedicated to further advancing this research and greatly appreciate the reviewer’s valuable feedback in shaping our future efforts.
